# Perturbation-theory machine learning for mood disorders: virtual design of dual inhibitors of NET and SERT proteins

**DOI:** 10.1186/s13065-024-01376-z

**Published:** 2025-01-02

**Authors:** Valeria V. Kleandrova, M. Natália D. S. Cordeiro, Alejandro Speck-Planche

**Affiliations:** LAQV@REQUIMTE/Department of Chemistry and Biochemistry, Faculty of Sciences, University of Porto, Porto, 4169-007 Portugal

**Keywords:** PTML, topological indices, Subgraph, fragment, fragment-based topological design, Multilayer perceptron, Mood disorders

## Abstract

Mood disorders affect the daily lives of millions of people worldwide. The search for more efficient therapies for mood disorders remains an active field of research. In silico approaches can accelerate the search for inhibitors against protein targets related to mood disorders. Here, we developed the first model perturbation-theory machine learning model based on a multiplayer perceptron network (PTML-MLP) for the simultaneous prediction and design of virtual dual-target inhibitors against two proteins associated with mood disorders, namely norepinephrine and serotonin transporters (NET and SERT, respectively). The PTML-MLP model had an accuracy of around 80%. From a chemical point of view, the PTML-MLP model could accurately identify both single- and dual-target inhibitors present in the dataset used to build it. Through the application of the fragment-based topological design (FBTD) approach, the molecular descriptors (multi-label graph-based indices) present in the PTML-MLP model were physicochemically and structurally interpreted. Such interpretations enabled (a) the extraction of different molecular fragments with a positive influence on the enhancement of the dual-target activity and (b) the design of four new drug-like molecules by assembling (fusing and/or connecting) several suitable molecular fragments. The designed molecules were predicted by the PTML-MLP model to exhibit dual-target activity against the NET and SERT proteins. These predictions, together with the estimated druglikeness suggest that the designed molecules could be new promising chemotypes to be considered for future synthesis and biological experimentation in the context of treatments for mood disorders.

**Supplementary Information:**

The online version contains supplementary material available at 10.1186/s13065-024-01376-z.

## Introduction

Mood disorders constitute complex and debilitating medical conditions, which are marked by disruptions in emotions. These include psychiatric illnesses such as major depressive disorder, hypomania, bipolar disorder, cyclothymia, and many others. Mood disorders are among the leading contributors to the global burden of diseases [1]. They affect around 20% of the population worldwide [2], and, according to a 2018 study reported in the USA, have an associated annual economic cost of more than $US326 billion [3]. However, over the past two decades, it has been demonstrated, that, for many patients with certain mood disorders, one medication may not be enough to tackle the broad range of symptoms they experience. For instance, 30% of patients with major depressive disorder will not remit even after going through separate courses of treatment with multiple antidepressants [4]. Generally speaking, standard treatments for mood disorders (e.g., antidepressants) have proven to be inefficient because the clinical conditions of the patients are either partially improved or remain completely unchanged. The lack of efficacy of the drugs used to treat mood disorders is closely linked to their pharmacology; they act through single mechanisms of action (by inhibiting only one target protein). Consequently, such drugs fall short because of the complex multi-genetic nature of the mood disorders [5]. This limitation, coupled with the serious side effects associated with the current treatments for mood disorders, has led to a reconsideration of the drug development strategies. Thus, there has been a paradigm shift characterized by the emergence of therapies based on dual-target or multi-target inhibitors, i.e., drugs/chemicals able to modulate more than one target involved in the appearance/progression of more or several types of mood disorders.

Before successfully developing an efficacious clinical treatment for mood disorders, it is essential to rationalize and speed up the search for novel and versatile chemicals with improved pharmacological profiles. Therefore, computational methods must be employed because they have become an essential part of most drug discovery campaigns [6]. In this context, many in silico approaches such as molecular docking [7, 8], network pharmacology [9], quantitative structure-activity relationships (QSAR) [10], pharmacophore modeling [11], and molecular dynamics simulation [12] have been used alone or in combination [11–15] for studying and/or discovering molecules that hold potential as future therapeutic solutions for mood disorders. Yet, these approaches, in addition to having the disadvantage of focusing on only one target protein, exhibit other drawbacks such as the use of structurally related series of molecules, the neglection of the impact of the different assay protocols on the activity values, and the lack of sufficiently clear physicochemical and structural interpretations. All these bottlenecks prevent the aforementioned computational methods from being used to design new molecules with desired (dual- or multi-target) pharmacological profiles against mood disorders.

During the last decade, the methodology known as perturbation theory combined with machine learning techniques (PTML) [16–18] has been developed to overcome the aforementioned issues present in the modern in silico approaches mentioned above. In doing so, by fusing chemical data with in vitro and/or in vivo biological information, PTML models have been successfully applied to different therapeutic areas such as oncology [19–23], infectious diseases [24–28], and neurosciences [29, 30]. At the same time, through the use of the fragment-based topological design (FBTD) approach, PTML models have been demonstrated to be interpretable [31, 32]. Thus, FBTD has enabled PTML models to be used as tools for the computer-aided design of novel molecules virtually exhibiting the desired biological activity [19, 27, 33].

To the best of our knowledge, there is no report of an in silico approach capable of enabling the discovery of dual- or multi-target inhibitors for the treatment of mood disorders. Such an approach would be beneficial because it would accelerate the early discovery of versatile chemicals with the potential to become clinically relevant treatments against mood disorders. Bearing in mind all the previous ideas, in this work, we have established the theoretical foundations for the rational discovery of new chemicals able to act as dual-target inhibitors against mood disorders. Particularly, we have combined a PTML model based on multilayer perceptron networks (PTML-MLP) with the FBTD approach to enable the in silico design and prediction of dual-target inhibitors against the proteins named norepinephrine and serotonin transporters (NET and SERT, respectively). The reason to choose NET and SERT is, that, from a pharmacological point of view, they are well-validated and attractive targets for the development of pharmacotherapeutic agents against mood disorders [34, 35].

## Materials and methods

### Dataset and molecular descriptors

**Fig. 1:**
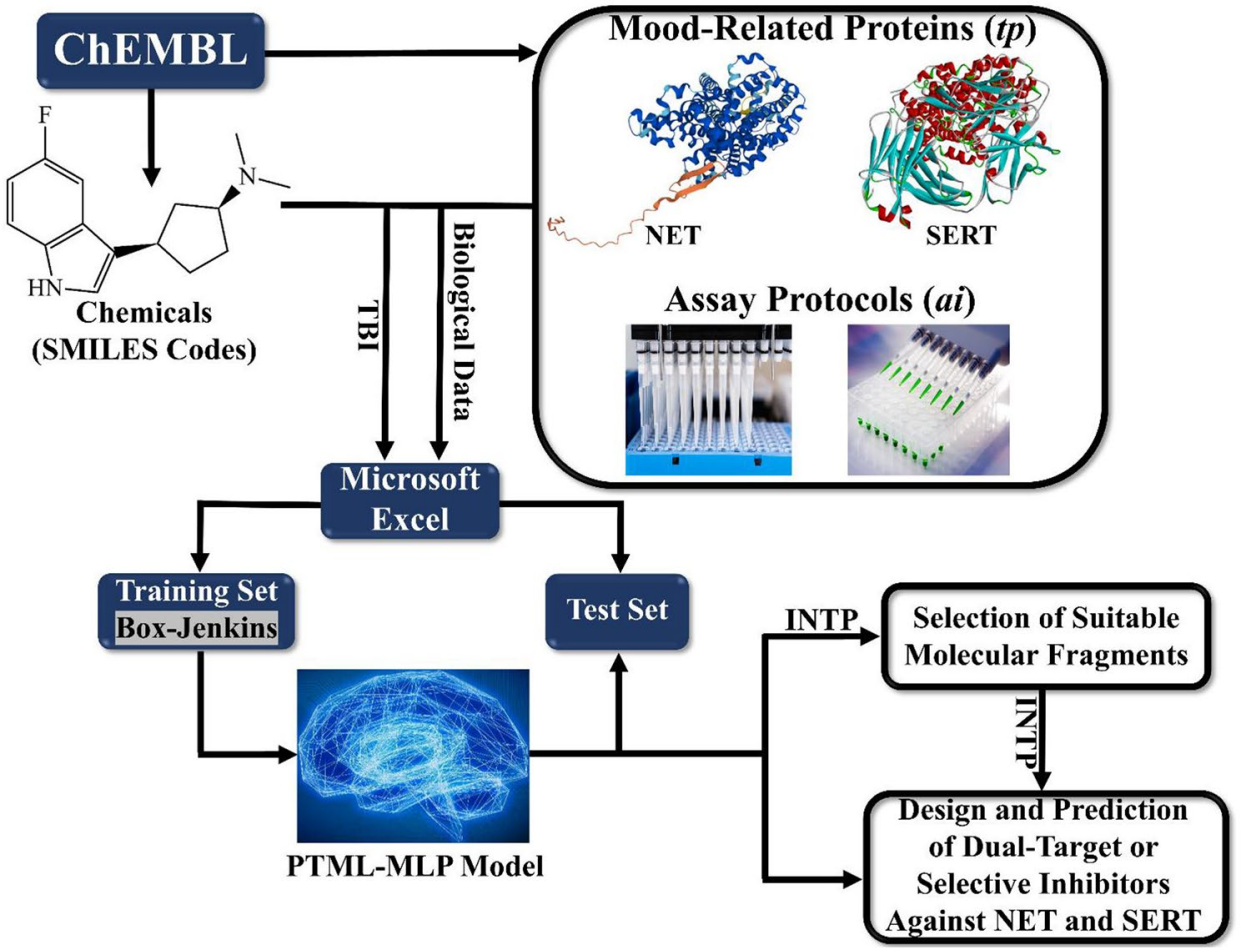
Developing a PTML-MLP model for the design of dual-target inhibitors of NET and SERT. Calculations associated with the Box-Jenkins approach are carried out using Microsoft Excel (see the first two equations mentioned below). The symbol “INTP” means the physicochemical and structural interpretation. Although the focus of the present research is to design and predict dual-target inhibitors, the PTML-MLP model developed in this work can also be used to predict selective inhibitors of either NET or SERT

All the stages involved in the creation of the PTML-MLP model have been described in detail very recently [28, 33, 36] and are also summarized in Fig. 1. Thus, we will only discuss here specific aspects which are different from previous reports. We retrieved all the chemical and biological data from the public web repository known as the ChEMBL database [37, 38]. The chemical data contained the identifiers and SMILES codes of each molecule while the biological data included the measures and values of inhibitory activity expressed as the half-maximal inhibitory concentration (IC_50_). Also, the retrieved data contained information on the target proteins and the assay protocols. We deleted all the entries with missing values or activity units, as well as those where the SMILES codes were absent. In the case of a potential duplicate (molecule tested more than one time against the same target protein and by considering the same assay protocol), we kept only the entry containing the lowest IC_50_ value.

We labeled each molecule as active [*IAi*(*cj*) = 1] if it exhibited IC_50_ ≤ 150 nM against a defined protein; otherwise, the molecule was annotated as inactive [*IAi*(*cj*) = − 1]. Notice that the cut-off value mentioned above was chosen because, apart from being a fairly rigorous activity value (low to medium nanomolar range), it allowed the dataset to be kept relatively balanced in terms of the number of active and inactive compounds. We would like to highlight that *IAi*(*cj*) was a categorical variable that indicated the inhibitory activity of the *i*th molecule under the experimental condition *cj*. At the same time, *cj* was formed by two elements, namely the target protein (*tp*) against which the assay was performed (NET or SERT) and the assay protocol (*ai*) employed to experimentally test the molecule.

We used the SMILES codes (stored in a txt file) as inputs to calculate the topological indices (*TIs*) known as the bond-based spectral moments. These were weighted by physicochemical properties such as hydrophobicity contributions (*Hyd*), polar surface areas (*Psa*), atomic refractivities (*Mol*), Gasteiger-Marsili charges (*Gas*), and atomic weights (*Ato*). We also calculated the atom- and bond-based connectivity indices. In this sense, the MODESLAB v1.5 software was employed for these calculations [39]. We also generated new descriptors *NTI* by dividing each *TI* value by *NB* (number of bonds without considering bond multiplicity). We then divided the dataset into training and test series (approximately 75% for the training set and 25% for the test set). In doing so, we sorted the molecules in increasing order of their IC_50_ values, assigning the first three molecules with the label “training” while annotating the fourth molecule to belong to the “test” set; this process was repeated for each protein separately. Following, we applied the Box-Jenkins moving average approach in two steps [40], which allowed us to merge the chemical data with the different aspects of the experimental information *cj* (*tp* and *ai*):

1 $$\:avg\left[TBI\right]cj=\frac{1}{n\left(cj\right)}\times\:\sum\:_{a=1}^{n\left(cj\right)}{TBI}_{a}$$

In Eq. 1, *TBI* refers to any *TI* or *NTI* descriptor mentioned above. As already explained in reference [40], the term *n*(*cj*) is the number of training cases (chemicals) labeled as active by considering the same element of the experimental condition *cj* (*tp* or *ai*). Also, *avg*[*TBI*]*cj* is an average value. We would like to highlight that Eq. 1 was applied to *tp* and *ai* separately [40]. In the second step, we calculated the multi-label indices *D*[*TBI*]*cj* for each chemical in the dataset:

2 $$\:D\left[TBI\right]cj=\left[\frac{TBI-avg\left[TBI\right]cj}{Std\left[TBI\right]}\right]\cdot\:\sqrt{p\left(cj\right)}$$

In Eq. 2, the terms *TBI* and *avg*[*TBI*]*cj* have been explained above. On the other hand, *Std*[*TBI*] is the standard deviation calculated from the *TBI* values by considering only the chemicals/cases present in the training set. Also, *p*(*cj*) was an a priori probability computed according to recent works [19, 40]. It is important to highlight that the multi-label graph-based indices *D*[*TBI*]*cj* are descriptors that measure how much a query molecule physicochemically and structurally deviates from a group of chemicals annotated as active and assayed under the same experimental conditions as that query molecule. As in the case of Eq. 1, we also applied Eq. 2 to the elements *tp* and *ai* separately. More details on the *TI*, *NTI*, *TBI*, *avg*[*TBI*]*cj*, *p*(*cj*), and *D*[*TBI*]*cj* values can be found in Supplementary Information 1.

### The PTML-MLP model: generation, performance, and applicability domain

Before generating the PTML-MLP model, we employed the IMMAN software (version 1.0) [41], which allowed us to calculate the information index known as the mutual information differential Shannon’s entropy (*MI-DSE*) [42]; the *D*[*TBI*]*cj* descriptors containing the greatest information contents (and, potentially, displaying the greatest discriminatory powers) were the ones exhibiting the highest *MI-DSE* values. We also assessed the level of redundancy among all the *D*[*TBI*]*cj* descriptors by computing Pearson’s correlation coefficient (*PCC*). The program used to compute the *PCC* values was STATISTICA v13.5.0.17 [43]; only the non-redundant *D*[*TBI*]*cj* descriptors (those complying with the conditions whose − 0.7 < *PCC* < + 0.7) were kept for further analysis. The artificial neural networks package of this same program was employed to search for the best MLP network (PTML-MLP model). In doing so, due to our experience in working with PTML-MLP models built from datasets of different sizes [19, 27, 44], as well as the fact that there were *T* = 2742 training cases in this work (see Supplementary Information 1), we used a predefined configuration for different tunable hyperparameters. One of them was the number of input nodes, which was set to be *I* = 15 (i.e., the fifteen non-redundant *D*[*TBI*]*cj* descriptors exhibiting the highest *MI-DSE* values). The number of output nodes was automatically set by STATISTICA v13.5.0.17 to be *O* = 2 because only two classes were predicted (active and inactive). Other tunable hyperparameters whose values were set arbitrarily according to our experience were the number of epochs (500), the minimum and maximum numbers of hidden neurons (with values of 15 and 70 respectively), the number of networks to train (3000), and the number of networks to be retained (250) [19, 27, 44]. Here, logistic, hyperbolic tangent, and exponential were chosen as the activation functions in both the hidden and the output layers. The best MLP network (PTML-MLP model) was the one exhibiting the highest values (in both training and test sets) of local and global sensitivities and specificities, as well as the normalized Matthew correlation coefficient (*nMCC*) [45]. Besides paying careful attention to all the aforementioned tunable hyperparameters, we wanted to prevent our PTML-MLP model from overfitting the data, and therefore, when searching for the best MLP network, we applied the following equation characterizing the network topology:

3 $$\:\rho\:=\frac{T}{\left[\right(I+1)H+(H+1\left)O\right]}$$

In Eq. 3*T*, *I*, and *O* have been defined above. In the case of *H*, this defines the number of hidden neurons. If *ρ* > 3, it can be assumed that the MLP network is not overfitting the data [46]. Last, the applicability domain (AD) of the PTML-MLP model was assessed according to a modification of the descriptor space’s approach reported by Speck-Planche and co-workers in recent works [19, 27, 44].

## Results and discussion

### The PTML-MLP model

The most appropriate PTML-MLP model found by us contained the notation MLP 15-26-2. This means that our PTML-MLP model has 15 nodes in the input layer, which is equivalent to the fifteen *D*[*TBI*]*cj* descriptors present in this model (Table 1). The hidden layer of the PTML-MLP model has 26 neurons; this layer uses hyperbolic tangent as the activation function. The notation also indicates that two categorical values (*IAi*(*cj*) = 1 indicating active and *IAi*(*cj*) = − 1 for inactive) were predicted by considering one node in the output layer; this layer used the activation function named identity.

**Table 1:** All the *D*[*TBI*]*cj* descriptors that entered in the PTML-MLP model

Symbol^a^	Code^b^	Definition^c^
*D*[*SM*(*Hyd*)1]*tp*	*DTBI*01	Spectral moment based on the hydrophobicity contributions of path subgraphs formed by one bond.
*D*[*SM*(*Mol*)2]*tp*	*DTBI*02	Spectral moment weighted by the molar refractivity in path subgraphs formed by two bonds.
*D*[*e*(*Ch*)6]*tp*	*DTBI*03	Bond connectivity index derived from chain (ring) subgraphs formed by six bonds.
*D*[*NX*(*P*)2]*tp*	*DTBI*04	Normalized atom connectivity index derived from path subgraphs formed by two bonds.
*D*[*NXv*(*P*)6]*tp*	*DTBI*05	Normalized valence connectivity index derived from path subgraphs formed by six bonds.
*D*[*Ne*(*P*)4]*tp*	*DTBI*06	Normalized bond connectivity index derived from path subgraphs formed by four bonds.
*D*[*SM*(*Hyd*)3]*ai*	*DTBI*07	Spectral moment based on the hydrophobicity contributions in cluster and/or chain (ring) subgraphs formed by three bonds.
*D*[*NSM*(*Psa*)1]*ai*	*DTBI*08	Normalized spectral moment weighted by the polar surface area in path subgraphs formed by one bond.
*D*[*NSM*(*Mol*)1]*ai*	*DTBI*09	Normalized spectral moment weighted by the molar refractivity in path subgraphs formed by one bond.
*D*[*NSM*(*Gas*)3]*ai*	*DTBI*10	Normalized spectral moment weighted by the Gasteiger-Marsili charges in cluster and/or chain (ring) subgraphs formed by three bonds.
*D*[*NX*(*P*)6]*ai*	*DTBI*11	Normalized atom connectivity index derived from path subgraphs formed by six bonds.
*D*[*NXv*(*C*)5]*ai*	*DTBI*12	Normalized valence connectivity index derived from cluster subgraphs formed by five bonds.
*D*[*Ne*(*P*)1]*ai*	*DTBI*13	Normalized bond connectivity index derived from path subgraphs formed by one bond.
*D*[*Ne*(*P*)2]*ai*	*DTBI*14	Normalized bond connectivity index derived from path subgraphs formed by two bonds.
*D*[*Ne*(*PC*)5]*ai*	*DTBI*15	Normalized bond connectivity index derived from path-cluster subgraphs formed by five bonds.

^a^Among all the *D*[*TBI*]*cj* descriptors, the ones ending with “*tp*” depend on both the chemical structure and the target protein against which a chemical was experimentally tested while the others ending with “*ai*” consider the chemical structure and the information regarding a defined assay protocol. ^b^Codes for the *D*[*TBI*]*cj* descriptors. Such codes will be used instead of symbols when analyzing the physicochemical and structural interpretations of the *D*[*TBI*]*cj* descriptors. ^c^The number of bonds in each fragment/subgraph is obtained without considering the bond multiplicity

If now we substitute the values of the parameters of the PTML-MLP model associated with the network topology (i.e., *T* = 2742, *I* = 15, *H* = 26, and *O* = 2 for training cases, input nodes, hidden neurons, and output nodes, respectively) in Eq. 3, we will obtain a value of *ρ* = 5.83. Since *ρ* > 3, it can be concluded that the PTML-MLP model is not overfitting the data [46, 47].

When considering the statistical performance, our PTML-EL-MLP model exhibited a fairly good accuracy of 86.32% in the training set. Also, the same statistical index had a value of 78.57% in the test set. Other statistical indices such as the number of active and inactive (*N*_Active_ and *N*_Inactive_, respectively), the number of cases correctly classified as active (*CC*_Active_) and inactive (*CC*_Active_), as well as the sensitivity (*Sn*), specificity (*Sp*), and normalized Matthew correlation coefficient (*nMCC*) values are depicted in Table 2.

**Table 2:** Global statistical quality and predicted power of the PTML-MLP model as well as other PTML models based on alternative supervised learning techniques

Symbols^a, b^	PTML-MLP		PTML-LDA		PTML-SVM		PTML-RF	
	Training Set	Test Set	Training Set	Test Set	Training Set	Test Set	Training Set	Test Set
*N*_Active_	1482	492	1482	492	1482	492	1482	492
*CC*_Active_	1317	401	925	312	1230	392	1243	390
*Sn*	88.87%	81.50%	62.42%	63.41%	83.00%	79.67%	83.87%	79.27%
*N*_Inactive_	1260	418	1260	418	1260	418	1260	418
*CC*_Inactive_	1050	314	774	251	938	265	1023	293
*Sp*	83.33%	75.12%	61.43%	60.05%	74.44%	63.40%	81.19%	70.10%
*nMCC*	0.862	0.784	0.619	0.617	0.789	0.719	0.825	0.748

^a^*N*_Active_ – Number of chemicals/cases annotated as active; *N*_Inactive_ – Number of chemicals/cases labeled as inactive; *CC*_Active_ – Chemicals/cases properly identified as active; *CC*_Inactive_ – Chemicals/cases properly identified as inactive; *Sn* – Sensitivity; *Sp* – Specificity; *nMCC* – Normalized Matthews correlation coefficient. ^b^The PTML models depicted here are the best found by us; the software used to find all the PTML models was STATISTICA v13.5.0.17. As in the case of the tunning hyperparameters reported by us for the PTML-MLP model (see Material and Methods section), the ones reported for the alternative PTML models are also provided. For PTML-LDA, the option of including all the *D*[*TBI*]*cj* descriptors was applied and the prior probability values for active and inactive were 0.485 and 0.515, respectively. The PTML-SVM model was obtained by using SVM classification type 2, radial basis functional as the kernel, gamma = 0.067, nu = 0.540, number of support vectors = 1623 (1336 bounded), number of iterations = 1000, and stopping error = 0.001. The PTML-RF found by us contained number of trees = 65, subsample proportion = 0.5, minimum number of cases = 40, maximum number of levels = 10, minimum number in the child node = 5, maximum number of nodes = 100, and prior probabilities of 0.475 and 0.525 for active and inactive, respectively

Notice that Table 2 offers a comparative analysis of the PTML-MLP model to other PTML models based on three different supervised learning techniques such as linear discriminant analysis (PTML-LDA), support vector machine (PTML-SVM), and random forest (PTML-RF). In this sense, as described in Table 2, all the PTML models were obtained using the same dataset (as indicated by *N*_Active_ and *N*_Inactive_). The PTML-LDA model had the worst performance. In the case of PTML-SVM, this model, despite exhibiting a relatively acceptable statistical quality (training set), its predictive power (test set) is greatly reduced since its *Sp* < 70%. The PTML-RF is the best among the three alternative models in terms of *Sn*, *Sp*, and *nMCC* values. However, the PTML-MLP model outperforms PTML-LDA, PTML-SVM, and PTML-RF, displaying the highest *Sn* and *Sp* values in both training and test sets. In the PTML-MLP model, *Sn* and *Sp* have values higher than 75%. Also, we would like to highlight that the *nMCC* can range from 0 (poor prediction) to 1 (ideal performance) while a value of 0.5 is associated with a random predictor. In the case of our PTML-MLP model, the *nMCC* values, besides being the closest to 1, are also the highest. This indicates that the PTML-MLP model has the strongest convergence between the observed [*IAi*(*cj*)] and the predicted [*PredIAi*(*cj*)] values of inhibitory activity. Altogether, the PTML-MLP model is better than the other three aforementioned alternative PTML models in terms of both statistical quality and predictive power (the classification results obtained by the PTML-MLP model for each case/chemical in our dataset can be found in Supplementary Material 2).

One of the benefits of our PTML-MLP model is that it can predict the inhibitory activity of chemicals against two proteins (NET and SERT) by considering more than one experimental condition *cj*. In this sense, Table 3 clearly shows that the PTML-MLP model considers a total of six different *cj* (which, as explained above, are combinations of the elements *tp* and *ai*), with four of them involving NET and the remaining two focused on SERT. This means that the PTML-MLP model can predict activity against any of these proteins in a consensus manner. For instance, a query molecule can be predicted four times against NET. If a molecule is predicted as active in at least 3 of the 4 conditions *cj*, then, that molecule can be considered as active. A similar line of thinking can be applied to SERT; if a query molecule is predicted as active in at least one of the two experimental conditions *cj*, then this molecule will be regarded as active.

**Table 3:** Summary of the diverse experimental conditions reported in the dataset used to create the PTML-MLP model

*cj* ^a^	*tp* ^b^	*ai* ^c^
*c*01	NET	B (single protein format)
*c*02	NET	B (cell-based format)
*c*03	NET	F (cell-based format)
*c*04	NET	B (cell membrane format)
*c*05	SERT	B (single protein format)
*c*06	SERT	B (cell-based format)

^a^The codes refer to the diverse experimental conditions *cj*, which are combinations of the elements *tp* and *ai* ^**b**^The target proteins associated with mood disorders, namely norepinephrine transporter (NET) and serotonin transporter (SERT). ^**c**^Assay information describing the combination of the columns “assay type” (first letter) and “bioassay ontology” (phrase between parentheses). Such columns were initially downloaded (together with the chemical and biological data) from the ChEMBL database and subsequently merged into column *a.i.* (assay information)

To support the capability of our PTML-MLP to perform the aforementioned consensus predictions, we calculated the values of the statistical metrics symbolized as [*Sn*(%)]*tp*, [*Sp*(%)]*tp*, [*Sn*(%)]*ai*, and [*Sp*(%)]*ai* These are the local counterparts of *Sn* and *Sp* but depend either on the target protein *tp* or the assay protocols *a.i.* Regardless of the training or test sets, the values for all these local metrics were higher than 70% (see Supplementary Material 2), which means that the PTML-MLP model can correctly classify/predict at least 70% of the chemicals (either active or inactive) across different targets and assays. We should say that the only exceptions were the [*Sn*(%)]*ai* values for assay “B (cell membrane format)”, which were 62.50% and 50.00% in training and test sets, respectively.

Although our PTML-MLP model can perform consensus predictions, we also assessed the AD, which, as mentioned in subsection 2.2, was carried out according to a variation of the descriptors’ space approach [27, 48]. Thus, we generated 15 local scores of applicability domain (one for each *D*[*TBI*]*cj* descriptor) by comparing each *D*[*TBI*]*cj* value of any query molecule with the corresponding maximum and minimum *D*[*TBI*]*cj* values. If the *D*[*TBI*]*cj* value of a molecule was inside the boundary formed by the maximum and minimum *D*[*TBI*]*cj* values, the local score took the value of one; otherwise, the local score took the value of zero. For each molecule present in the dataset used to build the PTML-MLP model, this procedure was applied to each of the *D*[*TBI*]*cj* descriptors. Then, we calculated the total score of the applicability domain (TSAD) as the summation of local scores. Because 15 *D*[*TBI*]*cj* descriptors are present in the PTML-MLP model, then, a molecule must have TSAD = 15 to belong to the AD of the PTML-MLP model. In our dataset, 3633 out of 3652 molecules/cases were within the AD of the PTML-MLP model (Supplementary Material 2); the deletion of the chemicals/cases outside the AD didn’t significantly affect the statistical performance of the PTML-MLP model.

**Fig. 2:**
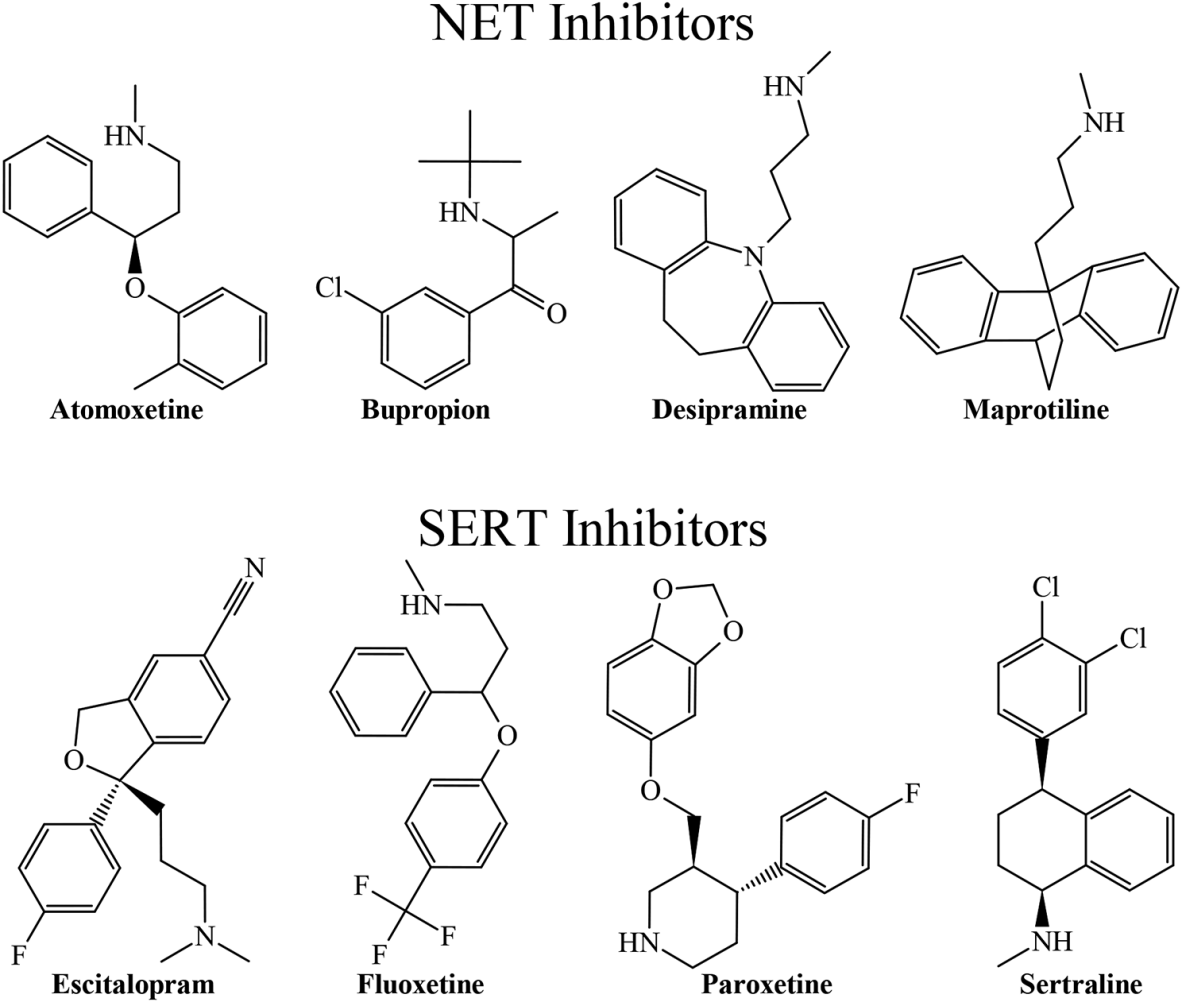
Selective inhibitors of either NET or SERT correctly predicted by the PTML-MLP model. Such drugs are atomoxetine (ChEMBL641), bupropion (ChEMBL894), desipramine (ChEMBL72), maprotiline (ChEMBL21731), escitalopram (ChEMBL1508), fluoxetine (ChEMBL41), paroxetine (ChEMBL490), and sertraline (ChEMBL809). They present a relatively great structural diversity, which indicates that our PTML-MLP model can identify selective inhibitors when performing virtual screening

Another advantage of the present PTML-MLP model is the ability to detect privileged molecular patterns. In doing so, our PTML-MLP model accurately predicted the inhibitory activity of well-established FDA-approved drugs for the treatment of mood disorders (Fig. 2) through selective inhibition of the proteins NET or SERT.

**Fig. 3:**
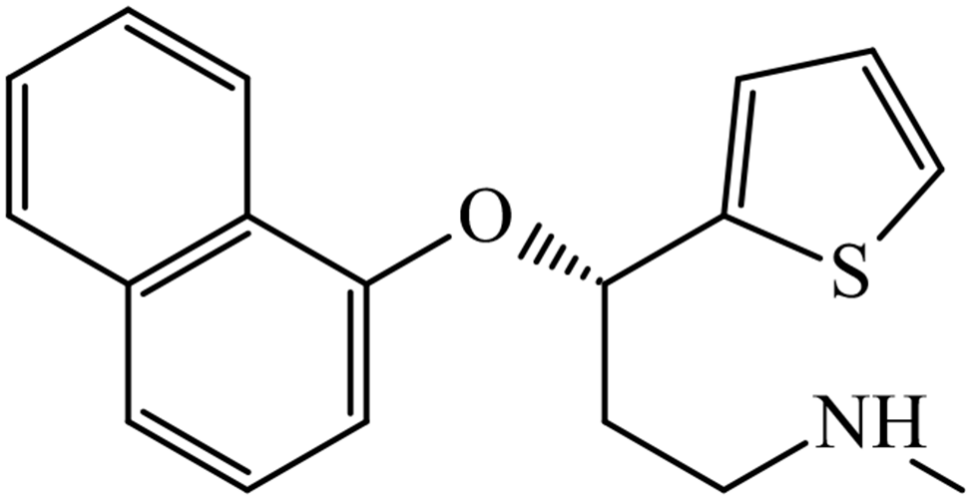
Chemical structure of duloxetine: a dual-target inhibitor of NET and SERT

At the same time, our PTML-MLP model was able to correctly predict the drug named duloxetine (ChEMBL1175). This chemical is a dual-target inhibitor of NET and SERT (Fig. 3) used to treat major depressive disorder and other medical conditions such as neuropathic pain, generalized anxiety disorder, and others.

The PTML-MLP also predicts other dual-target inhibitors such as chlorpromazine (ChEMBL71); however, based on the experimental IC_50_ values reported in the dataset used to build the PTML-MLP model, in terms of dual-target inhibition, chlorpromazine is considerably weaker than duloxetine. It can be seen that there are structural differences between duloxetine and the other selective inhibitors of NET and SERT mentioned above. In any case, the local metrics analyzed before, together with the illustrative examples discussed in Figs. 2 and 3, indicate the quality and capability of our PTML-MLP model in identifying both single- and dual-target inhibitors. The classification/prediction results of the drugs depicted in Figs. 2 and 3 can be found by searching the ChEMBL identifiers associated with each drug in Supplementary Material 2.

### Physicochemical and structural interpretation of the PTML-MLP model

**Fig. 4:**
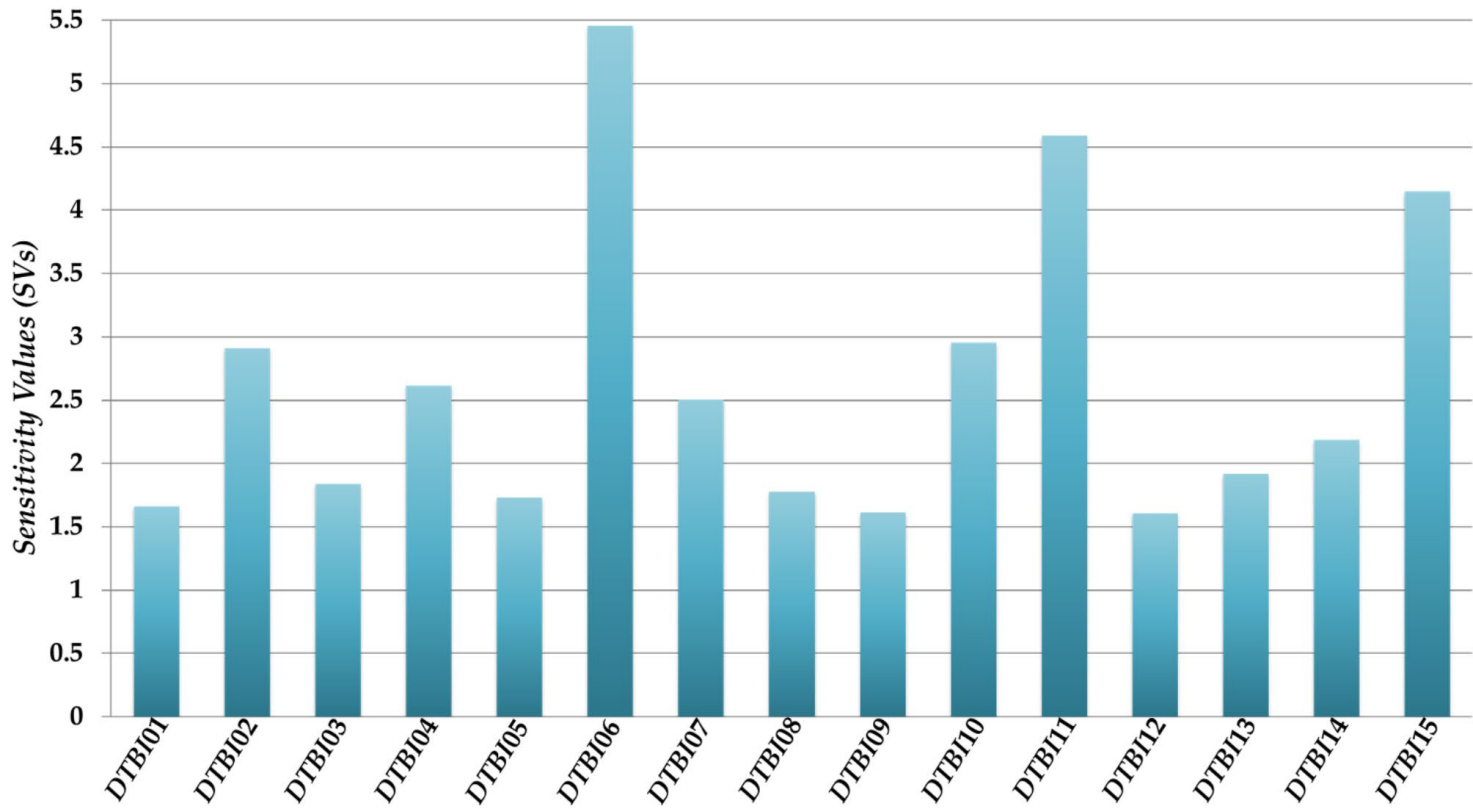
Relative importance of the *D*[*TBI*]*cj* descriptors in the PTML-MLP model measured by the sensitivity values (*SVs*). The symbols/codes appear according to the information found in Table 1

The application of the FBTD approach comprises two steps, (a) the physicochemical and structural interpretations of the *D*[*TBI*]*cj* descriptors present in the PTML-MLP model and (b) the design of new molecules using these interpretations as guidelines [27, 31, 49]. The purpose of this subsection was to apply the first of these steps to gather insights regarding the physicochemical properties and structural features that can be important for the appearance and/or enhancement of the dual-target activity of any chemical against NET and SERT. In this work, while interpreting the *D*[*TBI*]*cj* descriptors present in our PTML-MLP model, we have relied on their estimated sensitivity values (*SVs*), which are illustrated in Fig. 4.

We would like to emphasize that *SVs* quantify the relative importance of the input variables in a neural network model [50]. When applied to our PTML-MLP model, *SVs* rate the influence of the *D*[*TBI*]*cj* descriptors (input variables). On one side, the highest *SVs* are associated with those *D*[*TBI*]*cj* descriptors having the greatest relative influences (highest discriminatory powers) in the PTML-MLP model. On the other hand, from a more phenomenological point of view, the *D*[*TBI*]*cj* descriptors with the highest *SVs* are the ones whose physicochemical and structural information should be present in most of the molecules of the dataset used to build the PTML-MLP model. Such information is also essential for the future design of any new molecule with potential dual-target activity against NET and SERT proteins.

In addition to Fig. 4, we have applied a recently reported approach, which allowed us to calculate two class-based means for each *D*[*TBI*]*cj* descriptor (Table 4) from the cases/chemicals present in the training set [19, 27, 31, 48]. One of these means was determined by considering the chemicals correctly classified as active while the other was computed from the chemicals correctly identified as inactive. By comparing the two class-based means for each *D*[*TBI*]*cj* descriptor, we qualitatively estimated whether the value of a defined *D*[*TBI*]*cj* descriptor could be increased or decreased to enhance the activity against both NET and SERT.

**Table 4:** Propensity of variation of the *D*[*TBI*]*cj* descriptors

*D*[*TBI*]*cj* Descriptor^a^	Class-Based Means^b^		Propensity^c^
	Active	Inactive	
*DTBI*01	-2.3491 × 10^− 2^	1.7237 × 10^− 1^	Decrease
*DTBI*02	-2.0571 × 10^− 2^	2.3810 × 10^− 1^	Decrease
*DTBI*03	-2.4099 × 10^− 2^	2.9490 × 10^− 1^	Decrease
*DTBI*04	1.4380 × 10^− 2^	-2.0387 × 10^− 2^	Increase
*DTBI*05	-1.8983 × 10^− 2^	9.3305 × 10^− 2^	Decrease
*DTBI*06	-2.9150 × 10^− 2^	3.0225 × 10^− 2^	Decrease
*DTBI*07	-1.7325 × 10^− 2^	5.9041 × 10^− 2^	Decrease
*DTBI*08	-4.6074 × 10^− 3^	7.3880 × 10^− 2^	Decrease
*DTBI*09	2.1909 × 10^− 2^	-9.0758 × 10^− 2^	Increase
*DTBI*10	1.0287 × 10^− 2^	-1.3033 × 10^− 1^	Increase
*DTBI*11	-1.4183 × 10^− 2^	-1.3653 × 10^− 1^	Increase
*DTBI*12	3.2827 × 10^− 3^	-1.0293 × 10^− 1^	Increase
*DTBI*13	1.8964 × 10^− 3^	1.1222 × 10^− 1^	Decrease
*DTBI*14	1.4434 × 10^− 3^	1.4925 × 10^− 2^	Decrease
*DTBI*15	-1.0582 × 10^− 2^	-1.4020 × 10^− 1^	Increase

^a^The codes of the *D*[*TBI*]*cj* descriptors appear in the same order as in Table 1. ^b^Average values for each *D*[*TBI*]*cj* descriptor by considering the categories of active and inactive; only chemicals correctly classified in the training set by the PTML-MLP model were employed to calculate the class-based means. ^c^Propensity – Tendency of the value of each *D*[*TBI*]*cj* descriptor to be increased or decreased, being this associated with the enhancement of the dual-target activity against the mood disorder proteins NET and SERT

**Fig. 5:**
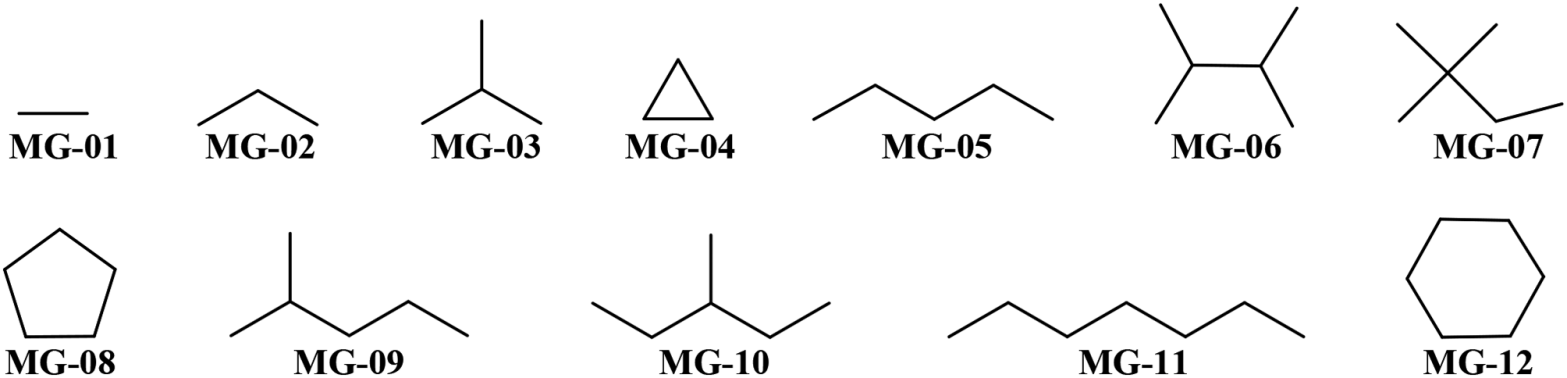
Subgraphs (generic fragments) whose presence is expected to desirably enhance the dual-target activity against NET and SERT

Also, when interpreting the *D*[*TBI*]*cj* descriptors of our PTML-MLP model, we have associated each of them with certain subgraphs/generic fragments. In doing so, we have also given examples of how these subgraphs can be present in the form of molecular fragments such as specific atoms, functional groups, rings, and/or substructural moieties whose presence positively contributes to the versatile activity [19, 27, 48], in this case, the dual-target activity against the proteins NET and SERT (Fig. 5).

It is essential to emphasize that when interpreting the *D*[*TBI*]*cj* descriptors, it must not be expected that their values should be increased or decreased infinitely. This comes from the fact that the values of the *D*[*TBI*]*cj* descriptors have their limits (given by the AD of the PTML-MLP model discussed above). At the same time, the physicochemical and structural information in a defined *D*[*TBI*]*cj* descriptor is constrained by one or more *D*[*TBI*]*cj* descriptors; consequently, the number of molecular fragments (derived from those subgraphs) is neither expected to vary (increase or decrease) infinitely.

In our PTML- MLP model, we have six *D*[*TBI*]*cj* descriptors derived from the bond-based spectral moments [51–56], whose increments in their values characterize the degree of concentration of different physicochemical properties in regions of diverse size in a molecule. Consequently, it is possible to know specific regions of the molecules that can act through either polar interactions (e.g., hydrogen or halogen bonding) or London dispersion forces. These descriptors are *DTBI*01, *DTBI*02, *DTBI*07, *DTBI*08, *DTBI*09, and *DTBI*10, and they rank thirteen, fifth, seventh, eleven, fourteen, and fourth among the most influential *D*[*TBI*]*cj* descriptors in the PTML-MLP model, respectively. In the case of *DTBI*01, this indicates the relative decrease of the global hydrophobicity of a molecule as the sum of the hydrophobic contributions of all the bonds in a molecule (MG-01 subgraphs). Also, according to *DTBI*02, the molar refractivity in the MG-02 subgraphs/fragments is expected to decrease, which means that the number of atoms with high polarizability (aromatic carbons, S, P, and halogens except fluorine) should be reduced. Consequently, aliphatic portions (including rings) are preferred over aromatic moieties. If aromatic carbons are present, they should be part of heteroaromatic rings such as pyridine, pyrazine, pyrimidine, imidazole, and/or oxazole; the last two rings are associated with subgraph MG-08 (which contains MG-02). When analyzing *DTBI*07, it can be seen that the hydrophobicity in the MG-03 and MG-04 subgraphs should be decreased. Therefore, the presence of functional groups such as amides, carboxyl, and sulfone groups, as well as three-membered heterocycles (e.g., oxirane, aziridine, and aziridin-2-one) will favorably decrease the value of *DTBI*07.

According to the information present in *DTBI*08, the global polar surface area (contributions of the MG-01 subgraphs) should decrease while increasing the total number of atoms. This means that the number of functional groups acting as hydrogen bond donors should be reduced as much as possible. The same is valid for the number of sulfur atoms. In the case of *DTBI*09, we can say that this *D*[*TBI*]*cj* descriptor constraints *DTBI*02 since the former expresses that the number of atoms with high polarizability (MG-01 subgraphs) should be increased while increasing the total number of atoms in a molecule. By jointly interpreting *DTBI*09 and *DTBI*02, we can deduce that the presence of at least one aromatic ring and several aliphatic portions is very important for the enhancement of the dual-target activity. Also, electronegative atoms such as N, O, and halogens (mainly F and Cl) should be distributed throughout the entire chemical structure of a molecule. The analysis of *DTBI*10 suggests an increment in the number of MG-03 and MG-04 subgraphs, which have aliphatic carbons (i.e., isopropyl group attached to any atom) or substructures containing electronegative atoms that aren’t bonded with hydrogens (e.g., N, N-dimethylamino, N,N-dialkyl amide, difluoromethyl, trifluoromethyl, cyclopropane, and oxirane).

In our PTML-MLP model, we also have *DTBI*03, *DTBI*06, *DTBI*13, *DTBI*14, and *DTBI*15; they are the tenth, first, ninth, eighth, and third most significant *D*[*TBI*]*cj* descriptors, respectively. These five graph-based variables are derived from the bond-based connectivity index. Therefore, they are measures of the molecular volume [57–59]. In the case of *DTBI*03, this expresses the diminution of the number of six-membered rings (subgraphs of the type MG-12). Our inspection of the dataset used to build the present PTML-MLP model indicates that the number of six-membered rings should not exceed three; these rings should preferably be polysubstituted. The volume should be diminished by decreasing the number of moieties that contain MG-05 subgraphs. This aspect is accounted for by *DTBI*06. Also, reducing the linearity of a molecule by adding a polysubstituted ring or more ramifications in the central part of a molecule will favorably decrease the value of *DTBI*06. A similar effect on the diminution of the volume is observed when analyzing the descriptors *DTBI*13 and *DTBI*14; while the former measures the average molecular volume (subgraph MG-01), the latter considers the same property in MG-02 subgraphs. In the case of *DTBI*15, this indicates the augmentation of the molecular volume by increasing the number of MG-07, MG-09, and MG-10 subgraphs. Examples of specific functional groups and substructural moieties containing these fragments are trifluoromethyl, N,N-dialkylamino, and any other group (or atom) attached to any atom within a ring. We would like to highlight that MG-07, MG-09, and MG-10 can also refer to aliphatic portions.

The remaining four *D*[*TBI*]*cj* descriptors are derived from the atom-based connectivity index, and thus, they constitute measures of molecular accessibility [60, 61], i.e., the ability of different regions in a molecule to be available to interact with the surrounding medium. The *D*[*TBI*]*cj* descriptors with such information content are *DTBI*04, *DTBI*05, *DTBI*11, and *DTBI*12. These rank sixth, twelfth, second, and fifteenth among the most relevant descriptors in the PTML-MLP model, respectively. The information contained in *DTBI*04 implies an increase in the molecular accessibility in MG-02 subgraphs, where the presence of more than one methyl group and/or halogen in the periphery of the molecule is a highly beneficial factor. In the case of *DTBI*05, the favorable diminution of the value of this descriptor indicates the augmentation of the number of atoms able to form hydrogen bonds in MG-11 subgraphs. This means that within each MG-11 subgraph, at least two electronegative atoms (N and O) should be present. The information contained in *DTBI*05 is in some way constrained by the one present in *DTBI*11, i.e., in the latter, either the number of MG-11 subgraphs should be increased or the number of methyl groups and halogen atoms should be augmented. Last, *DTBI*12 characterizes the increase of MG-06 subgraphs, and examples of substructural moieties containing MG-06 are the fused ring systems and N, N-dialkyl amides, as well as the dimethyl amino, difluoromethyl, and trifluoromethyl groups attached to any ring.

### Virtual design of dual-target inhibitors of NET and SERT

In this section, we applied the second step of the FBTD approach, which focused on using the joint physicochemical and structural interpretations of the *D*[*TBI*]*cj* descriptors as guidelines to design new molecules. The joint interpretation of the *D*[*TBI*]*cj* descriptors in the PTML-MLP model allowed us to consider the final characteristics that a molecule should possess to exhibit dual-target activity. Such characteristics are summarized as follows. Aliphatic portions and rings can appear in any part of a molecule. Any designed molecule should have a maximum of three (di- or trisubstituted) six-membered rings, with all of them being preferably located in the periphery of the molecule. Also, two of these three six-membered rings should be aliphatic. The central part of the molecule should contain either small ramifications (with each of them having a maximum of two bonds) or a polysubstituted five-membered heteroaromatic ring. The presence of at least two methyl groups (each of them attached to an electronegative atom such as nitrogen or oxygen) and/or at least one halogen (particularly Cl attached to an aromatic ring) in the periphery of the molecule can be a favorable factor. When possible, OHs and NHs should be avoided; if present, the sum of OHs and NHs should not exceed two. Electronegative atoms such as nitrogen and oxygen should be separated by two or more bonds (without counting bond multiplicity). A simple inspection of the dataset used to develop our PTML-MLP model suggests that the molecular weight of the designed molecules should not surpass 450 Da.

**Fig. 6:**
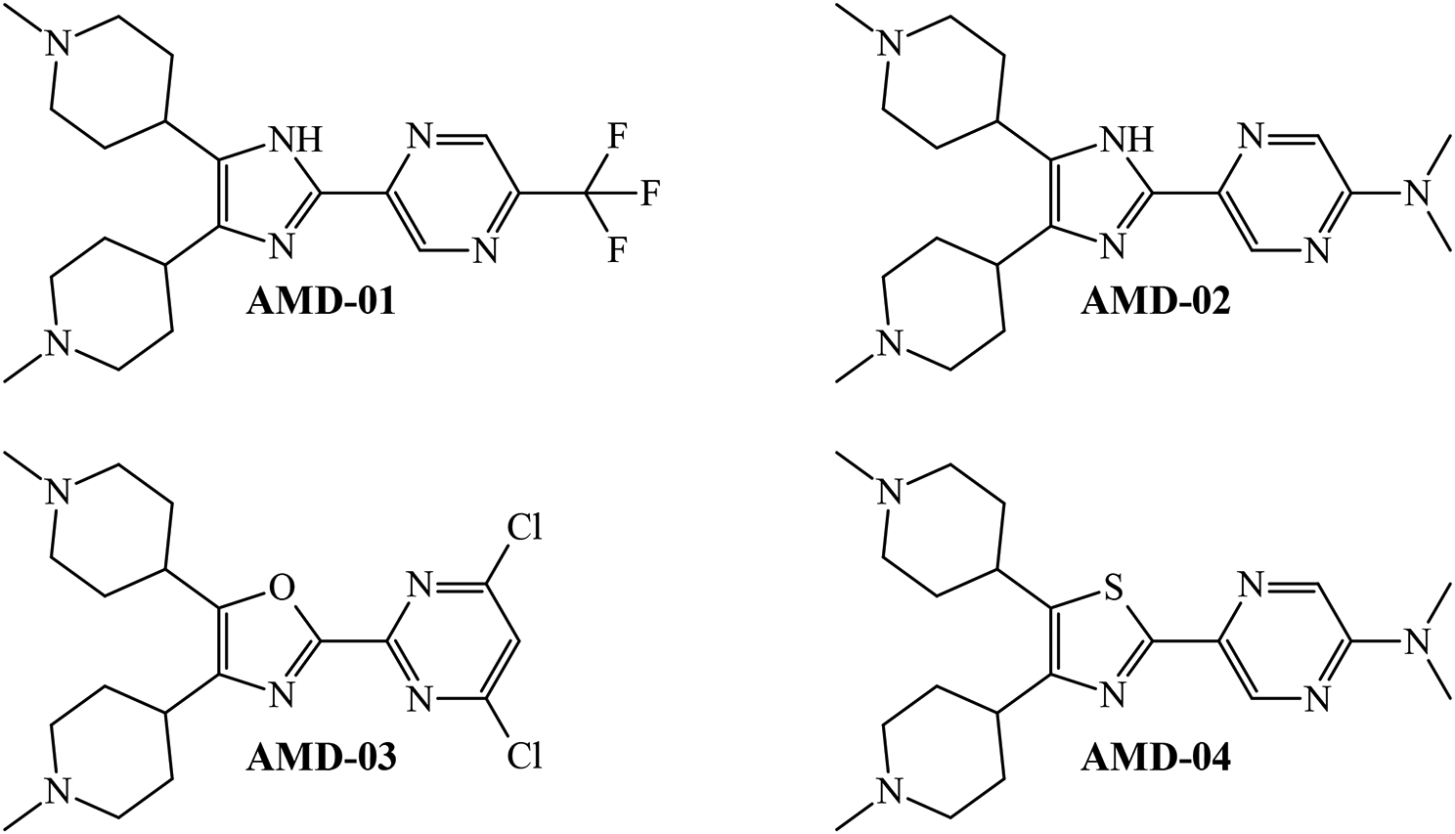
Molecules designed as virtual dual-target inhibitors against NET and SERT

We connected and/or fused different subgraph-based molecular fragments (e.g., functional groups, rings, and moieties derived from the subgraphs mentioned above). As a result, four molecules were designed (Fig. 6). It is important to highlight that the subgraph-based molecular fragments used to design the four molecules complied with the condition that their presence was beneficial for the favorable variation (increase or decrease) of the value of more than one *D*[*TBI*]*cj* descriptor in the PTML-MLP model.

A summary of the predictions performed by the PTML-MLP model regarding the activity profile of the designed molecules appears in Table 5 while all the details can be found in Supplementary Material 3.

**Table 5:** The designed molecules and their predicted dual-target activities

PREDICTIONS^a, b,c, d^							
ID	*cj*	*PredIAi(cj)*	*ProbAct(%)*	ID	*cj*	*PredIAi(cj)*	*ProbAct(%)*
AMD-01	*c*01	1	66.32	AMD-03	*c*01	1	62.06
AMD-01	*c*02	-1	45.16	AMD-03	*c*02	1	62.87
AMD-01	*c*03	1	81.34	AMD-03	*c*03	1	81.98
AMD-01	*c*04	-1	30.67	AMD-03	*c*04	-1	35.10
AMD-01	*c*05	1	70.80	AMD-03	*c*05	1	50.68
AMD-01	*c*06	-1	47.31	AMD-03	*c*06	1	56.54
AMD-02	*c*01	1	57.73	AMD-04	*c*01	1	69.69
AMD-02	*c*02	1	75.61	AMD-04	*c*02	1	84.38
AMD-02	*c*03	1	79.94	AMD-04	*c*03	1	61.36
AMD-02	*c*04	-1	39.77	AMD-04	*c*04	1	65.81
AMD-02	*c*05	-1	40.66	AMD-04	*c*05	-1	35.44
AMD-02	*c*06	1	63.58	AMD-04	*c*06	1	55.30

^a^ID – Identifier for each designed molecule. ^b^*cj* – The experimental conditions, which have been reported in the same order as in Table 3. ^c^*PredIAi*(*cj*) – Predicted categorical value of activity against a specific mood-related protein (*tp*) and by considering a defined assay protocol (*ai*). ^d^*ProbAct*(%) – Probability (expressed in percentage) predicted by the PTML-MLP model for a molecule to be active

The numbers represented in Table 5 are the predicted probabilities for each designed molecule to be identified as active under each of the six experimental conditions reported here (see Table 3). We considered a molecule to have dual-target inhibitory activity if its predicted probability was higher than 50% in at least 2 of 4 and at least 1 of 2 experimental conditions *cj* for NET and SERT, respectively. Based on this criterion, we can say that all the designed molecules behave as dual-target inhibitors. Molecule AMD-01 was the one being predicted in a lower number of experimental conditions *cj* (3 of the 6). In this sense, we would like to highlight that although AMD-01 is very similar to AMD-02, with the former having a trifluoromethyl group in position 5 of the pyrazine ring and the latter containing an N, N-dimethylamino group in the same position. This means that the N, N-dimethylamino group seems to be more appropriate since it favorably decreases the local and global hydrophobicity (explained by the descriptors *DTBI*01 and *DTBI*07, respectively) while increasing the ability of the molecule to interact through London dispersion forces (characterized by the *DTBI*12). In general, from the molecule AMD-02 to AMD-04, chemical modifications such as the replacement of certain heteroatoms (e.g., replacement of the pyrrolic nitrogen by oxygen or sulfur) or heterocycles in positions 2, 4, and 5 of the central five-membered rings, can either maintain or improve the dual-target activity. As depicted in Table 5, among the designed molecules, the most suitable chemical is AMD-03 because it was predicted as active in 5 of the 6 experimental conditions *cj*. A great contributor to the dual-target activity of AMD-03 is the fragment 4,6-dichloropyrimidine. Considering other fragments in the same position and comparing the values of all the *D*[*TBI*]*cj* descriptors among the designed molecules, the 4,6-dichloropyrimidine fragment gives AMD-03 the best balance of hydrophobicity, polarizability, and molecular volume. Consequently, the 4,6-dichloropyrimidine fragment in AMD-03 should be able to interact more effectively through London dispersion forces (aromatic carbons and chlorine) and polar interactions (e.g., hydrogen bonds through the pyridinic nitrogen atoms).

The previous subsections have shown that our PTML-MLP model, when combined with the FBTD approach, can be used to design molecules potentially exhibiting dual-target inhibition against NET and SERT. In any case, we wanted to assess the structural novelty of the designed molecules. Here, our purpose was to gain insights into whether the molecules designed in this work could represent new molecular entities. To do so, we performed a search in prestigious databases such as ChEMBL [37, 38, 62], ZINC [63], eMolecules [64], and SureChEMBL [65]. When searching in the aforementioned databases to find molecules similar to the ones designed by us, we used a similarity cutoff of 80%; under this condition, we could confirm that there were no molecules whose chemical structures resembled the ones designed in this work. This demonstrates that the joint use of our PTML model and the FBTD approach enables the generation of molecules, which, besides virtually exhibiting dual-target activity against NET and SERT, also constitute new chemotypes for future synthesis and biological evaluation in the context of mood disorders.

### Druglikeness and ADMET properties of the designed molecules

In addition to the novelty and the promising dual-target potency predicted for the designed molecules by the PTML-MLP model, we wanted to assess their druglikeness and properties related to their absorption, distribution, metabolism, elimination, and toxicity (ADMET) profiles. In this sense, we calculated several global physicochemical properties (Table 6).

**Table 6:** Physicochemical properties calculated for the designed molecules

ID^a^	MW	NHBA	NHBD	MlogP	AlogP	NAT	AMR	NRB	TPSA
AMD-01	408.53	8	1	2.396	2.822	56	105.43	3	60.94
AMD-02	383.61	6	1	2.339	2.152	61	114.57	4	64.18
AMD-03	410.39	6	0	3.366	3.404	52	107.82	3	58.29
AMD-04	400.66	6	0	2.286	2.72	60	118.76	4	76.63

^a^The following symbols are: MW – molecular weight (expressed in daltons); NHBA – number of hydrogen bond acceptors; NHBD – number of hydrogen bond donors; MlogP – logarithm of the partition coefficient (octanol/water) estimated according to Moriguchi’s approach; AlogP – logarithm of the partition coefficient (octanol/water) estimated according to Ghose-Crippen’s group contribution model; NAT – number of atoms; AMR – molar refractivity (expressed in cm^3^/mol) based on the Ghose-Crippen’s group contribution model; NRB – number of rotatable bonds; TPSA – topological polar (expressed in Å^2^) surface area considering nitrogen, oxygen, sulfur, and phosphorus

We employed the software AlvaDesc v1.0.22 [66] to perform the aforementioned calculations. The idea here was to compare the estimated values of the physicochemical properties of the designed molecules with the corresponding cutoff values established by Lipinski’s rule of five [67], Ghose’s filter [68], and Veber’s rules [69]. The analysis of the results in Table 6 indicates that the four designed molecules comply with the three druglikeness-based guidelines, indicating that they are likely to have acceptable oral bioavailability.

On the other hand, we used the systematic evaluation module of the web server named ADMETLab [70], which allowed us to calculate 31 pharmacokinetic and toxicity endpoints for each of the designed molecules (Supplementary Material 4). Summarizing, regarding the absorption, acceptable values of Papp (Caco-2) permeability, human intestinal absorption, and oral bioavailability were reported for the four designed molecules. In terms of distribution, the molecules were predicted to exhibit values of plasma protein binding (PPB) lower than 85%, as well as volumes of distribution in the desirable range (0.04–20 L/kg). It should be highlighted that the four designed molecules exhibited adequate blood-brain barrier (BBB) permeability. Notice that BBB permeability is a very important property for those chemicals aiming to act on the central nervous system as is the case of the drugs for the treatment of mood disorders. When analyzing the metabolism, the designed molecules exhibited moderate to low levels of promiscuity when predicted against the five major cytochromes P450 (CYP) enzymes, namely CYP1A2, CYP3A4, CYP2C9, CYP2C19, and CYP2D6. In general, the designed molecules were predicted as poor inhibitors of these CYPs, while also being estimated mainly as substrates of CYP1A2, CYP3A4, and CYP2C19. Regarding the elimination profiles, the designed molecules were predicted to exhibit low clearance. In terms of toxicity, a property of concern is that the designed molecules may behave as hERG blockers. However, regarding this toxic profile, the developers of the web server used the rigorous cutoff value IC_50_ < 40 µM (usually, the cutoff for off-target toxicity is IC_50_ ≤ 10 µM). Therefore, it cannot be concluded that our designed molecules could exhibit cardiotoxicity due to hERG inhibition. On the other hand, although a certain degree of hepatotoxicity was predicted for the designed molecules, it was also estimated that they would not cause drug-induced liver injury. In the case of mutagenicity, skin sensitization, and in vivo acute toxicity, the designed molecules (except AMD-01 predicted to exhibit certain in vivo toxicity) were predicted as safe chemicals. Altogether, the relatively acceptable pharmacokinetic and toxicity profiles predicted by ADMETLab web server indicate that the molecules designed by us in this work deserve further exploration at the experimental level in the context of early drug discovery for mood disorders.

## Conclusions

Mood disorders are a group of complex medical conditions whose treatment based on single-target drugs has proven to be ineffective. New chemicals used as dual- or multi-target therapeutic agents could provide better efficacy. Our PTML-MLP model created in this work has been a promising attempt to accelerate the discovery of chemicals with the potential to tackle mood disorders by acting as dual-target inhibitors of the NET and SERT proteins. When compared to other works reported in the field, our study provides deeper insights regarding the physicochemical aspects and structural features which can be essential for the appearance and enhancement of the dual-target activity against the aforementioned proteins. In the context of treatments for mood disorders, our PTML-MLP model is beneficial for basic science because it can accelerate the early discovery of novel chemicals with dual-target activity. However, due to its good predictive power, our PTML-MLP model could also prove useful in translational research as a tool for drug repurposing, thus identifying FDA-approved drugs with different therapeutic indications that have the potential to treat mood disorders. The present work opens new horizons on the development and application of interpretable machine learning models in different therapeutic areas.

## Electronic supplementary material

Below is the link to the electronic supplementary material.

Supplementary Material 1: *D[TBI]cj* descriptors, classification results, local metrics, and applicability domain.

Supplementary Material 2: Topological descriptors (*TIs* and *NTIs*), *D[TBI]cj* descriptors, classification results, and applicability domain for the designed molecules.

Supplementary Material 3: Topological descriptors (*TIs* and *NTIs*), averages *avg*[*TBI*]*cj*, and standard deviation values *Std*[*TBI*].

Supplementary Material 4

## Data Availability

Data is provided within the manuscript and supplementary material files.
