# Supplementary material for: Perturbation-theory machine learning for mood disorders: virtual design of dual inhibitors of NET and SERT proteins
Source: BMC Chem. 2025 Jan 2;19(1):2. doi: 10.1186/s13065-024-01376-z (PMC11697510; doi:10.1186/s13065-024-01376-z)
Supplement: Supplementary file 4 — Supplementary Material 4 [file 13065_2024_1376_MOESM4_ESM.pdf]

Perturbation-Theory Machine Learning for Mood Disorders: Virtual Design of Dual Inhibitors of NET and SERT Proteins

Valeria V. Kleandrova[1], M. Natália D. S. Cordeiro[1], and Alejandro Speck-Planche[1,*]

[1] *LAQV@REQUIMTE/Department of Chemistry and Biochemistry, Faculty of Sciences, University of Porto, 4169-007, Porto, Portugal*

***Corresponding Author:** Alejandro Speck-Planche (alejspivanovich@gmail.com);

ORCID ID: Alejandro Speck-Planche (https://orcid.org/0000-0002-9544-9016).
Valeria V. Kleandrova (https://orcid.org/0000-0002-1928-853X).
M. Natália D. S. Cordeiro (https://orcid.org/0000-0003-3375-8670).

The present supplementary material file contains the different pharmacokinetic and toxicity endpoints predicted for the four molecules designed in this work. These endpoints were estimated by the web server named ADMETLab.

Molecule AMD-01

| Query molecule

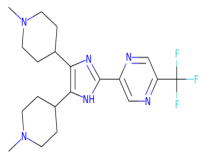

SMILES	CN1CCC(CC1)c1c(C2CCN(C)CC2)nc(c2cnc(C(F)(F)F)cn2)[nH]1
Molecular Weight	408.472
Log P (Crippen method)	3.022
HB Acceptor	5
HB Donor	1
TPSA	60.94

| Results

Physicochemical Property

Property	Predicted values	Suggestions	Meaning & Preference	Reference
LogS (Solubility)	-4.026 log mol/L (38.474 µg/mL)	> 10 µg/ml	• Optimal: higher than -4 log mol/L<br>• <10 µg/mL: Low solubility; 10–60 µg/mL: Moderate solubility; >60 µg/mL: High solubility	• Book: ISBN: 9787562832287. pp. 14<br>• J PHARMACOL TOX MET. 2000, 44 (1), 235–249;
$LogD_{7.4}$ (Distribution Coefficient D)	2.529	1~5	• < 1: Solubility high; Permeability low by passive transcellular diffusion; Permeability possible via paracellular if MW < 200; Metabolism low.<br>• 1 to 3: Solubility moderate; Permeability moderate; Metabolism low.<br>• 3 to 5: Solubility low; Permeability high; Metabolism moderate to high.<br>• > 5: Solubility low; Permeability high; Metabolism high.	• Methods and principles in medicinal chemistry 18 (pp. 21–45). Weinheim: Wiley-VCH.

Property	Predicted values	Suggestions	Meaning & Preference	Reference
LogP (Distribution Coefficient P)	3.022	0~3	• Optimal: 0< LogP <3<br>• LogP <0: poor lipid bilayer permeability.<br>• LogP >3: poor aqueous solubility.	• Book: ISBN: 3-906390-22-5. pp. 127–182.

Absorption

Property	Predicted values	Probability	Suggestions	Meaning & Preference	Reference
Papp (Caco-2 Permeability)	-4.637 cm/s		> -5.15 cm/s	Optimal: higher than -5.15 Log unit or -4.70 or -4.80	• J CHEM INF MODEL. 2016, 56 (4), pp 763–773.
Pgp-inhibitor	+	0.596		• The Pgp-inhibitor & non-inhibitor classification criteria refers the reference.	• J CHEM INF MODEL. 2010. 50(6): p. 1034-1041.<br>• J MED CHEM. 2011. 54(6): p. 1740-1751.
Pgp-substrate	-	0.401		• More likely to be a Pgp substrate: N+O ≥ 8; MW > 400; Acid with pKa > 4<br>• More likely to be a Pgp non-substrate: N+O ≤ 4; MW < 400; Acid with pKa < 8	• J DRUG TARGET. 11, 391–406.
HIA (Human Intestinal Absorption)	++	0.812		• ≥30%: HIA+; <30%: HIA-	• RSC ADV. 2017, 7, 19007-19018
F (20% Bioavailability)	++	0.75		• ≥20%: F20+; <20%: F20-	• MOL PHARMACEUT, 2011. 8(3): p. 841-851<br>• J PHARMACEUT BIOMED, 2008. 47(4): p. 677-682.
F (30% Bioavailability)	+	0.678		• ≥30%: F30+; <30%: F30-	• MOL PHARMACEUT, 2011. 8(3): p. 841-851<br>• J PHARMACEUT BIOMED, 2008. 47(4): p. 677-682.

Distribution

Property	Predicted values	Probability	Suggestions	Meaning & Preference	Reference
PPB (Plasma Protein Binding)	72.278 %		90%	• Significant with drugs that are highly protein-bound and have a low therapeutic index.	• ISBN: 978-0-1236-9520-8. pp. 194

Property	Predicted values	Probability	Suggestions	Meaning & Preference	Reference
VD (Volume Distribution)	0.424 L/kg		0.04~20 L/kg	• Optimal: 0.04-20L/kg;<br>• Range:<br><0.07L/kg: Confined to blood, Bound to plasma protein or highly hydrophilic; 0.07-0.7L/kg: Evenly distributed; >0.7L/kg: Bound to tissue components (e.g., protein, lipid),highly lipophilic.	• Book: ISBN: 9787562832287. pp. 174<br>• Book: ISBN: 978-0-1236-9520-8. pp. 229
BBB (Blood–Brain Barrier)	+++	0.937		• BB ratio >=0.1: BBB+; BB ratio <0.1: BBB-<br>• These features tend to improve BBB permeation:<br>H-bonds (total) < 8–10; MW < 400–500; No acids.	• J NEUROCHEM. 70, 1781–1792

Metabolism

Property	Predicted values	Probability	Meaning & Preference	Reference
P450 CYP1A2 inhibitor	---	0.147	• Molecules that labeled inhibitor in PubChem BioAssay were regarded as inhibitor.	• NAT BIOTECHNOL. 2009, 27(11): 1050-1055.<br>• BIOINFORMATICS. 2013, 29(16): 2051-2052.
P450 CYP1A2 Substrate	+	0.526	• Molecules that labeled substrate in PubChem BioAssay were regarded as substrate.<br>• Characteristics of CYP1A2 substrate: 0.08< LogP <3.61; Planar amines and amides	• NAT BIOTECHNOL. 2009, 27(11): 1050-1055.<br>• BIOINFORMATICS. 2013, 29(16): 2051-2052.
P450 CYP3A4 inhibitor	---	0.051	• Molecules that labeled inhibitor in PubChem BioAssay were regarded as inhibitor.<br>• Strategies to Reduce CYP3A4 Inhibition: Decrease the lipophilicity ($LogD_{7.4}$); Add steric hindrance to the heterocycle para to the nitrogen; Add an electronic substitution (e.g., halogen) that reduces the pKa of the nitrogen.	• NAT BIOTECHNOL. 2009, 27(11): 1050-1055.<br>• BIOINFORMATICS. 2013, 29(16): 2051-2052.
P450 CYP3A4 substrate	+	0.556	• Molecules that labeled substrate in PubChem BioAssay were regarded as substrate.<br>• Characteristics of CYP3A4 substrate: 0.97< LogP <7.54; Large molecules	• NAT BIOTECHNOL. 2009, 27(11): 1050-1055.<br>• BIOINFORMATICS. 2013, 29(16): 2051-2052.<br>• ISBN: 978-0-1236-9520-8. pp. 162

Property	Predicted values	Probability	Meaning & Preference	Reference
P450 CYP2C9 inhibitor	---	0.086	• Molecules that labeled inhibitor in PubChem BioAssay were regarded as inhibitor.	• NAT BIOTECHNOL. 2009, 27(11): 1050-1055. • BIOINFORMATICS. 2013, 29(16): 2051-2052.
P450 CYP2C9 substrate	-	0.403	• Molecules that labeled substrate in PubChem BioAssay were regarded as substrate. • Characteristics of CYP2C9 substrate: 0.89< LogP <5.18; Acidic (Nonionized)	• MOL INFORM. 2011. 30(10): p. 885-895. • J CHEM INF MODEL. 2013. 53(12): p. 3373-3383. • ISBN: 978-0-1236-9520-8. pp. 162
P450 CYP2C19 inhibitor	---	0.272	• Molecules that labeled inhibitor in PubChem BioAssay were regarded as inhibitor.	• NAT BIOTECHNOL. 2009, 27(11): 1050-1055. • BIOINFORMATICS. 2013, 29(16): 2051-2052.
P450 CYP2C19 substrate	+	0.529	• Molecules that labeled substrate in PubChem BioAssay were regarded as substrate.	• NAT BIOTECHNOL. 2009, 27(11): 1050-1055. • BIOINFORMATICS. 2013, 29(16): 2051-2052.
P450 CYP2D6 inhibitor	-	0.42	• Molecules that labeled inhibitor in PubChem BioAssay were regarded as inhibitor.	• MOL INFORM. 2011. 30(10): p. 885-895. • J CHEM INF MODEL. 2013. 53(12): p. 3373-3383.
P450 CYP2D6 substrate	-	0.432	• Molecules that labeled substrate in PubChem BioAssay were regarded as substrate. • Characteristics of CYP2D6 substrate: 0.75< LogP <5.04; Basic (Ionized)	• MOL INFORM. 2011. 30(10): p. 885-895. • J CHEM INF MODEL. 2013. 53(12): p. 3373-3383. • ISBN: 978-0-1236-9520-8. pp. 162

Elimination

Property	Predicted values	Suggestions	Meaning & Preference	Reference
$T_{1/2}$ (Half Life Time)	1.913 h	> 0.5 h	• Range: >8h: high; 3h< Cl < 8h: moderate; <3h: low	• ISBN: 978-0-1236-9520-8. pp. 236
CL (Clearance Rate)	1.571 mL/min/kg		• Range: >15 mL/min/kg: high; 5mL/min/kg< Cl < 15mL/min/kg: moderate; <5 mL/min/kg: low	• ISBN: 978-0-1236-9520-8. pp. 236

Toxicity

Property	Predicted values	Probability	Suggestions	Meaning & Preference	Reference
hERG (hERG Blockers)	++	0.856		• Where molecules with IC50 < 40 μM were regarded as blockers.<br>• Features may lead to hERG blocker:<br>◦ A basic amine (positively ionizable, pKa >7.3).<br>◦ Hydrophobic/lipophilic substructure(s) (ClogP >3.7).<br>◦ Absence of negatively ionizable groups or oxygen H-bond acceptors.	• TRENDS PHARMACOL SCI. 2005, 26(3): 119-124<br>• ISBN: 978-0-1236-9520-8. pp. 213<br>• MOL PHARM. 2016, 13(8):2855–2866
H-HT (Human Hepatotoxicity)	+	0.58		• The H-HT positive(+) & negative(-) classification criteria refers the reference.	• CHEM RES TOXICOL, 2016, 29(5): 757-767.
AMES (Ames Mutagenicity)	---	0.184		• Ames positive(+) & negative(-): significantly induces revertant colony growth at least in one out of usually five strains, otherwise, negative.	• J CHEM INF MODEL. 2012, 52(11): 2840-2847.
SkinSen (Skin sensitization)	-	0.323		• Sensitizer & Non-sensitizer: The (r)LLNA experimental value. (r)LLNA: (Reduced) local lymph node assay.	• TOXICOL APPL PHARM, 2015 , 284 (2) :262-272
LD50 (LD50 of acute toxicity)	3.637 -log mol/kg (94.224 mg/kg)		> 500 mg/kg	• Median lethal dose (LD50) usually represents the acute toxicity of chemicals.It is the dose amount of a tested molecule to kill 50 % of the treated animals within a given period.<br>• High-toxicity: 1~50 mg/kg; Toxicity: 51~500 mg/kg; low-toxicity: 501~5000 mg/kg.	• CHEM RES TOXICOL, 2009, 22 (12), pp 1913–1921<br>• J CHEMINFORMATICS, 2016 , 8 (1) :6
DILI (Drug Induced Liver Injury)	-	0.33		• The DILI positive(+) & negative(-) classification criteria refers the reference.	• J CHEM INF MODEL, 2015, 55(10) :2085-2093
FDAMDD (Maximum Recommended Daily Dose)	-	0.322		• The FDAMDD positive(+) & negative(-) classification criteria refers the reference.	• CHEMOMETR INTELL LAB, 2015, 146:494-502

Molecule AMD-02

| Query molecule

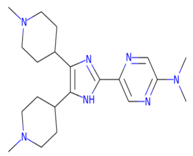

SMILES	CN1CCC(CC1)c1c(C2CCN(C)CC2)nc(c2cnc(N(C)C)cn2)[nH]1
Molecular Weight	383.544
Log P (Crippen method)	2.069
HB Acceptor	6
HB Donor	1
TPSA	64.18

| Results

Physicochemical Property

Property	Predicted values	Suggestions	Meaning & Preference	Reference
LogS (Solubility)	-3.185 log mol/L (250.504 µg/mL)	> 10 µg/ml	• Optimal: higher than -4 log mol/L<br>• <10 µg/mL: Low solubility; 10–60 µg/mL: Moderate solubility; >60 µg/mL: High solubility	• Book: ISBN: 9787562832287. pp. 14<br>• J PHARMACOL TOX MET. 2000, 44 (1), 235–249;
$LogD_{7.4}$ (Distribution Coefficient D)	2.163	1~5	• < 1: Solubility high; Permeability low by passive transcellular diffusion; Permeability possible via paracellular if MW < 200; Metabolism low.<br>• 1 to 3: Solubility moderate; Permeability moderate; Metabolism low.<br>• 3 to 5: Solubility low; Permeability high; Metabolism moderate to high.<br>• > 5: Solubility low; Permeability high; Metabolism high.	• Methods and principles in medicinal chemistry 18 (pp. 21–45). Weinheim: Wiley-VCH.

Property	Predicted values	Suggestions	Meaning & Preference	Reference
LogP (Distribution Coefficient P)	2.069	0~3	• Optimal: 0< LogP <3<br>• LogP <0: poor lipid bilayer permeability.<br>• LogP >3: poor aqueous solubility.	• Book: ISBN: 3-906390-22-5. pp. 127–182.

Absorption

Property	Predicted values	Probability	Suggestions	Meaning & Preference	Reference
Papp (Caco-2 Permeability)	-4.668 cm/s		> -5.15 cm/s	Optimal: higher than -5.15 Log unit or -4.70 or -4.80	• J CHEM INF MODEL. 2016, 56 (4), pp 763–773.
Pgp-inhibitor	-	0.488		• The Pgp-inhibitor & non-inhibitor classification criteria refers the reference.	• J CHEM INF MODEL. 2010. 50(6): p. 1034-1041.<br>• J MED CHEM. 2011. 54(6): p. 1740-1751.
Pgp-substrate	++	0.791		• More likely to be a Pgp substrate: N+O ≥ 8; MW > 400; Acid with pKa > 4<br>• More likely to be a Pgp non-substrate: N+O ≤ 4; MW < 400; Acid with pKa < 8	• J DRUG TARGET. 11, 391–406.
HIA (Human Intestinal Absorption)	++	0.718		• ≥30%: HIA+; <30%: HIA-	• RSC ADV. 2017, 7, 19007-19018
F (20% Bioavailability)	++	0.715		• ≥20%: F20+; <20%: F20-	• MOL PHARMACEUT, 2011. 8(3): p. 841-851<br>• J PHARMACEUT BIOMED, 2008. 47(4): p. 677-682.
F (30% Bioavailability)	+	0.636		• ≥30%: F30+; <30%: F30-	• MOL PHARMACEUT, 2011. 8(3): p. 841-851<br>• J PHARMACEUT BIOMED, 2008. 47(4): p. 677-682.

Distribution

Property	Predicted values	Probability	Suggestions	Meaning & Preference	Reference
PPB (Plasma Protein Binding)	61.741 %		90%	• Significant with drugs that are highly protein-bound and have a low therapeutic index.	• ISBN: 978-0-1236-9520-8. pp. 194

Property	Predicted values ❓	Probability ❓	Suggestions ❓	Meaning & Preference	Reference
VD (Volume Distribution)	1.043 L/kg		0.04~20 L/kg	• Optimal: 0.04-20L/kg;<br>• Range:<br><0.07L/kg: Confined to blood, Bound to plasma protein or highly hydrophilic; 0.07-0.7L/kg: Evenly distributed; >0.7L/kg: Bound to tissue components (e.g., protein, lipid),highly lipophilic.	• Book: ISBN: 9787562832287. pp. 174<br>• Book: ISBN: 978-0-1236-9520-8. pp. 229
BBB (Blood–Brain Barrier)	++	0.735		• BB ratio >=0.1: BBB+; BB ratio <0.1: BBB-<br>• These features tend to improve BBB permeation:<br>H-bonds (total) < 8–10; MW < 400–500; No acids.	• J NEUROCHEM. 70, 1781–1792

Metabolism

Property	Predicted values ❓	Probability ❓	Meaning & Preference	Reference
P450 CYP1A2 inhibitor	---	0.18	• Molecules that labeled inhibitor in PubChem BioAssay were regarded as inhibitor.	• NAT BIOTECHNOL. 2009, 27(11): 1050-1055.<br>• BIOINFORMATICS. 2013, 29(16): 2051-2052.
P450 CYP1A2 Substrate	+	0.558	• Molecules that labeled substrate in PubChem BioAssay were regarded as substrate.<br>• Characteristics of CYP1A2 substrate: 0.08< LogP <3.61; Planar amines and amides	• NAT BIOTECHNOL. 2009, 27(11): 1050-1055.<br>• BIOINFORMATICS. 2013, 29(16): 2051-2052.
P450 CYP3A4 inhibitor	---	0.041	• Molecules that labeled inhibitor in PubChem BioAssay were regarded as inhibitor.<br>• Strategies to Reduce CYP3A4 Inhibition: Decrease the lipophilicity ($LogD_{7.4}$); Add steric hindrance to the heterocycle para to the nitrogen; Add an electronic substitution (e.g., halogen) that reduces the pKa of the nitrogen.	• NAT BIOTECHNOL. 2009, 27(11): 1050-1055.<br>• BIOINFORMATICS. 2013, 29(16): 2051-2052.
P450 CYP3A4 substrate	+	0.602	• Molecules that labeled substrate in PubChem BioAssay were regarded as substrate.<br>• Characteristics of CYP3A4 substrate: 0.97< LogP <7.54; Large molecules	• NAT BIOTECHNOL. 2009, 27(11): 1050-1055.<br>• BIOINFORMATICS. 2013, 29(16): 2051-2052.<br>• ISBN: 978-0-1236-9520-8. pp. 162

Property	Predicted values	Probability	Meaning & Preference	Reference
P450 CYP2C9 inhibitor	---	0.058	• Molecules that labeled inhibitor in PubChem BioAssay were regarded as inhibitor.	• NAT BIOTECHNOL. 2009, 27(11): 1050-1055.<br>• BIOINFORMATICS. 2013, 29(16): 2051-2052.
P450 CYP2C9 substrate	-	0.361	• Molecules that labeled substrate in PubChem BioAssay were regarded as substrate.<br>• Characteristics of CYP2C9 substrate: 0.89< LogP <5.18; Acidic (Nonionized)	• MOL INFORM. 2011. 30(10): p. 885-895.<br>• J CHEM INF MODEL. 2013. 53(12): p. 3373-3383.<br>• ISBN: 978-0-1236-9520-8. pp. 162
P450 CYP2C19 inhibitor	---	0.04	• Molecules that labeled inhibitor in PubChem BioAssay were regarded as inhibitor.	• NAT BIOTECHNOL. 2009, 27(11): 1050-1055.<br>• BIOINFORMATICS. 2013, 29(16): 2051-2052.
P450 CYP2C19 substrate	+	0.64	• Molecules that labeled substrate in PubChem BioAssay were regarded as substrate.	• NAT BIOTECHNOL. 2009, 27(11): 1050-1055.<br>• BIOINFORMATICS. 2013, 29(16): 2051-2052.
P450 CYP2D6 inhibitor	-	0.456	• Molecules that labeled inhibitor in PubChem BioAssay were regarded as inhibitor.	• MOL INFORM. 2011. 30(10): p. 885-895.<br>• J CHEM INF MODEL. 2013. 53(12): p. 3373-3383.
P450 CYP2D6 substrate	-	0.437	• Molecules that labeled substrate in PubChem BioAssay were regarded as substrate.<br>• Characteristics of CYP2D6 substrate: 0.75< LogP <5.04; Basic (Ionized)	• MOL INFORM. 2011. 30(10): p. 885-895.<br>• J CHEM INF MODEL. 2013. 53(12): p. 3373-3383.<br>• ISBN: 978-0-1236-9520-8. pp. 162

Elimination

Property	Predicted values	Suggestions	Meaning & Preference	Reference
$T_{1/2}$ (Half Life Time)	1.817 h	> 0.5 h	• Range: >8h: high; 3h< Cl < 8h: moderate; <3h: low	• ISBN: 978-0-1236-9520-8. pp. 236
CL (Clearance Rate)	1.886 mL/min/kg		• Range: >15 mL/min/kg: high; 5mL/min/kg< Cl < 15mL/min/kg: moderate; <5 mL/min/kg: low	• ISBN: 978-0-1236-9520-8. pp. 236

Toxicity

Property	Predicted values	Probability	Suggestions	Meaning & Preference	Reference
hERG (hERG Blockers)	++	0.742		• Where molecules with IC50 < 40 µM were regarded as blockers.<br>• Features may lead to hERG blocker:<br>◦ A basic amine (positively ionizable, pKa >7.3).<br>◦ Hydrophobic/lipophilic substructure(s) (ClogP >3.7).<br>◦ Absence of negatively ionizable groups or oxygen H-bond acceptors.	• TRENDS PHARMACOL SCI. 2005, 26(3): 119-124<br>• ISBN: 978-0-1236-9520-8. pp. 213<br>• MOL PHARM. 2016, 13(8):2855–2866
H-HT (Human Hepatotoxicity)	-	0.48		• The H-HT positive(+) & negative(-) classification criteria refers the reference.	• CHEM RES TOXICOL, 2016, 29(5): 757-767.
AMES (Ames Mutagenicity)	---	0.216		• Ames positive(+) & negative(-): significantly induces revertant colony growth at least in one out of usually five strains, otherwise, negative.	• J CHEM INF MODEL. 2012, 52(11): 2840-2847.
SkinSen (Skin sensitization)	-	0.335		• Sensitizer & Non-sensitizer: The (r)LLNA experimental value. (r)LLNA: (Reduced) local lymph node assay.	• TOXICOL APPL PHARM, 2015 , 284 (2) :262-272
LD50 (LD50 of acute toxicity)	2.724 -log mol/kg (724.128 mg/kg)		> 500 mg/kg	• Median lethal dose (LD50) usually represents the acute toxicity of chemicals.It is the dose amount of a tested molecule to kill 50 % of the treated animals within a given period.<br>• High-toxicity: 1~50 mg/kg; Toxicity: 51~500 mg/kg; low-toxicity: 501~5000 mg/kg.	• CHEM RES TOXICOL, 2009, 22 (12), pp 1913–1921<br>• J CHEMINFORMATICS, 2016 , 8 (1) :6
DILI (Drug Induced Liver Injury)	-	0.48		• The DILI positive(+) & negative(-) classification criteria refers the reference.	• J CHEM INF MODEL, 2015, 55(10) :2085-2093
FDAMDD (Maximum Recommended Daily Dose)	-	0.352		• The FDAMDD positive(+) & negative(-) classification criteria refers the reference.	• CHEMOMETR INTELL LAB, 2015, 146:494-502

Molecule AMD-03

| Query molecule

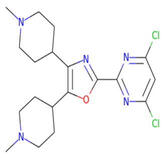

SMILES	CN1CCC(CC1)c1c(C2CCN(C)CC2)nc(c2nc(Cl)cc(Cl)n2)o1
Molecular Weight	410.349
Log P (Crippen method)	4.057
HB Acceptor	6
HB Donor	0
TPSA	58.29

| Results

Physicochemical Property

Property	Predicted values	Suggestions	Meaning & Preference	Reference
LogS (Solubility)	-4.857 log mol/L (5.704 µg/mL)	> 10 µg/ml	• Optimal: higher than -4 log mol/L<br>• <10 µg/mL: Low solubility; 10–60 µg/mL: Moderate solubility; >60 µg/mL: High solubility	• Book: ISBN: 9787562832287. pp. 14<br>• J PHARMACOL TOX MET. 2000, 44 (1), 235–249;
$LogD_{7.4}$ (Distribution Coefficient D)	2.989	1~5	• < 1: Solubility high; Permeability low by passive transcellular diffusion; Permeability possible via paracellular if MW < 200; Metabolism low.<br>• 1 to 3: Solubility moderate; Permeability moderate; Metabolism low.<br>• 3 to 5: Solubility low; Permeability high; Metabolism moderate to high.<br>• > 5: Solubility low; Permeability high; Metabolism high.	• Methods and principles in medicinal chemistry 18 (pp. 21–45). Weinheim: Wiley-VCH.

Property	Predicted values	Suggestions	Meaning & Preference	Reference
LogP (Distribution Coefficient P)	4.057	0~3	• Optimal: 0< LogP <3<br>• LogP <0: poor lipid bilayer permeability.<br>• LogP >3: poor aqueous solubility.	• Book: ISBN: 3-906390-22-5. pp. 127–182.

Absorption

Property	Predicted values	Probability	Suggestions	Meaning & Preference	Reference
Papp (Caco-2 Permeability)	-4.647 cm/s		> -5.15 cm/s	Optimal: higher than -5.15 Log unit or -4.70 or -4.80	• J CHEM INF MODEL. 2016, 56 (4), pp 763–773.
Pgp-inhibitor	++	0.754		• The Pgp-inhibitor & non-inhibitor classification criteria refers the reference.	• J CHEM INF MODEL. 2010. 50(6): p. 1034-1041.<br>• J MED CHEM. 2011. 54(6): p. 1740-1751.
Pgp-substrate	---	0.264		• More likely to be a Pgp substrate: N+O ≥ 8; MW > 400; Acid with pKa > 4<br>• More likely to be a Pgp non-substrate: N+O ≤ 4; MW < 400; Acid with pKa < 8	• J DRUG TARGET. 11, 391–406.
HIA (Human Intestinal Absorption)	++	0.814		• ≥30%: HIA+; <30%: HIA-	• RSC ADV. 2017, 7, 19007-19018
F (20% Bioavailability)	++	0.754		• ≥20%: F20+; <20%: F20-	• MOL PHARMACEUT, 2011. 8(3): p. 841-851<br>• J PHARMACEUT BIOMED, 2008. 47(4): p. 677-682.
F (30% Bioavailability)	+	0.66		• ≥30%: F30+; <30%: F30-	• MOL PHARMACEUT, 2011. 8(3): p. 841-851<br>• J PHARMACEUT BIOMED, 2008. 47(4): p. 677-682.

Distribution

Property	Predicted values	Probability	Suggestions	Meaning & Preference	Reference
PPB (Plasma Protein Binding)	72.916 %		90%	• Significant with drugs that are highly protein-bound and have a low therapeutic index.	• ISBN: 978-0-1236-9520-8. pp. 194

Property	Predicted values	Probability	Suggestions	Meaning & Preference	Reference
VD (Volume Distribution)	0.437 L/kg		0.04~20 L/kg	• Optimal: 0.04-20L/kg;<br>• Range:<br><0.07L/kg: Confined to blood, Bound to plasma protein or highly hydrophilic; 0.07-0.7L/kg: Evenly distributed; >0.7L/kg: Bound to tissue components (e.g., protein, lipid),highly lipophilic.	• Book: ISBN: 9787562832287. pp. 174<br>• Book: ISBN: 978-0-1236-9520-8. pp. 229
BBB (Blood–Brain Barrier)	+++	0.979		• BB ratio >=0.1: BBB+; BB ratio <0.1: BBB-<br>• These features tend to improve BBB permeation:<br>H-bonds (total) < 8–10; MW < 400–500; No acids.	• J NEUROCHEM. 70, 1781–1792

Metabolism

Property	Predicted values	Probability	Meaning & Preference	Reference
P450 CYP1A2 inhibitor	-	0.436	• Molecules that labeled inhibitor in PubChem BioAssay were regarded as inhibitor.	• NAT BIOTECHNOL. 2009, 27(11): 1050-1055.<br>• BIOINFORMATICS. 2013, 29(16): 2051-2052.
P450 CYP1A2 Substrate	+	0.576	• Molecules that labeled substrate in PubChem BioAssay were regarded as substrate.<br>• Characteristics of CYP1A2 substrate: 0.08< LogP <3.61; Planar amines and amides	• NAT BIOTECHNOL. 2009, 27(11): 1050-1055.<br>• BIOINFORMATICS. 2013, 29(16): 2051-2052.
P450 CYP3A4 inhibitor	---	0.112	• Molecules that labeled inhibitor in PubChem BioAssay were regarded as inhibitor.<br>• Strategies to Reduce CYP3A4 Inhibition: Decrease the lipophilicity ($LogD_{7.4}$); Add steric hindrance to the heterocycle para to the nitrogen; Add an electronic substitution (e.g., halogen) that reduces the pKa of the nitrogen.	• NAT BIOTECHNOL. 2009, 27(11): 1050-1055.<br>• BIOINFORMATICS. 2013, 29(16): 2051-2052.
P450 CYP3A4 substrate	+	0.616	• Molecules that labeled substrate in PubChem BioAssay were regarded as substrate.<br>• Characteristics of CYP3A4 substrate: 0.97< LogP <7.54; Large molecules	• NAT BIOTECHNOL. 2009, 27(11): 1050-1055.<br>• BIOINFORMATICS. 2013, 29(16): 2051-2052.<br>• ISBN: 978-0-1236-9520-8. pp. 162

Property	Predicted values	Probability	Meaning & Preference	Reference
P450 CYP2C9 inhibitor	---	0.061	• Molecules that labeled inhibitor in PubChem BioAssay were regarded as inhibitor.	• NAT BIOTECHNOL. 2009, 27(11): 1050-1055.<br>• BIOINFORMATICS. 2013, 29(16): 2051-2052.
P450 CYP2C9 substrate	---	0.299	• Molecules that labeled substrate in PubChem BioAssay were regarded as substrate.<br>• Characteristics of CYP2C9 substrate: 0.89< LogP <5.18; Acidic (Nonionized)	• MOL INFORM. 2011. 30(10): p. 885-895.<br>• J CHEM INF MODEL. 2013. 53(12): p. 3373-3383.<br>• ISBN: 978-0-1236-9520-8. pp. 162
P450 CYP2C19 inhibitor	---	0.148	• Molecules that labeled inhibitor in PubChem BioAssay were regarded as inhibitor.	• NAT BIOTECHNOL. 2009, 27(11): 1050-1055.<br>• BIOINFORMATICS. 2013, 29(16): 2051-2052.
P450 CYP2C19 substrate	+	0.646	• Molecules that labeled substrate in PubChem BioAssay were regarded as substrate.	• NAT BIOTECHNOL. 2009, 27(11): 1050-1055.<br>• BIOINFORMATICS. 2013, 29(16): 2051-2052.
P450 CYP2D6 inhibitor	-	0.45	• Molecules that labeled inhibitor in PubChem BioAssay were regarded as inhibitor.	• MOL INFORM. 2011. 30(10): p. 885-895.<br>• J CHEM INF MODEL. 2013. 53(12): p. 3373-3383.
P450 CYP2D6 substrate	-	0.495	• Molecules that labeled substrate in PubChem BioAssay were regarded as substrate.<br>• Characteristics of CYP2D6 substrate: 0.75< LogP <5.04; Basic (Ionized)	• MOL INFORM. 2011. 30(10): p. 885-895.<br>• J CHEM INF MODEL. 2013. 53(12): p. 3373-3383.<br>• ISBN: 978-0-1236-9520-8. pp. 162

Elimination

Property	Predicted values	Suggestions	Meaning & Preference	Reference
$T_{1/2}$ (Half Life Time)	1.701 h	> 0.5 h	• Range: >8h: high; 3h< Cl < 8h: moderate; <3h: low	• ISBN: 978-0-1236-9520-8. pp. 236
CL (Clearance Rate)	1.573 mL/min/kg		• Range: >15 mL/min/kg: high; 5mL/min/kg< Cl < 15mL/min/kg: moderate; <5 mL/min/kg: low	• ISBN: 978-0-1236-9520-8. pp. 236

Toxicity

Property	Predicted values	Probability	Suggestions	Meaning & Preference	Reference
hERG (hERG Blockers)	++	0.86		• Where molecules with IC50 < 40 µM were regarded as blockers.<br>• Features may lead to hERG blocker:<br>◦ A basic amine (positively ionizable, pKa >7.3).<br>◦ Hydrophobic/lipophilic substructure(s) (ClogP >3.7).<br>◦ Absence of negatively ionizable groups or oxygen H-bond acceptors.	• TRENDS PHARMACOL SCI. 2005, 26(3): 119-124<br>• ISBN: 978-0-1236-9520-8. pp. 213<br>• MOL PHARM. 2016, 13(8):2855–2866
H-HT (Human Hepatotoxicity)	+	0.55		• The H-HT positive(+) & negative(-) classification criteria refers the reference.	• CHEM RES TOXICOL, 2016, 29(5): 757-767.
AMES (Ames Mutagenicity)	---	0.228		• Ames positive(+) & negative(-): significantly induces revertant colony growth at least in one out of usually five strains, otherwise, negative.	• J CHEM INF MODEL. 2012, 52(11): 2840-2847.
SkinSen (Skin sensitization)	-	0.37		• Sensitizer & Non-sensitizer: The (r)LLNA experimental value. (r)LLNA: (Reduced) local lymph node assay.	• TOXICOL APPL PHARM, 2015 , 284 (2) :262-272
LD50 (LD50 of acute toxicity)	2.573 -log mol/kg (1096.866 mg/kg)		> 500 mg/kg	• Median lethal dose (LD50) usually represents the acute toxicity of chemicals.It is the dose amount of a tested molecule to kill 50 % of the treated animals within a given period.<br>• High-toxicity: 1~50 mg/kg; Toxicity: 51~500 mg/kg; low-toxicity: 501~5000 mg/kg.	• CHEM RES TOXICOL, 2009, 22 (12), pp 1913–1921<br>• J CHEMINFORMATICS, 2016 , 8 (1) :6
DILI (Drug Induced Liver Injury)	-	0.45		• The DILI positive(+) & negative(-) classification criteria refers the reference.	• J CHEM INF MODEL, 2015, 55(10) :2085-2093
FDAMDD (Maximum Recommended Daily Dose)	-	0.376		• The FDAMDD positive(+) & negative(-) classification criteria refers the reference.	• CHEMOMETR INTELL LAB, 2015, 146:494-502

Molecule AMD-04

| Query molecule

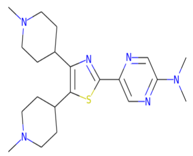

SMILES	CN1CCC(CC1)c1c(C2CCN(C)CC2)nc(c2cnc(N(C)C)cn2)s1
Molecular Weight	400.596
Log P (Crippen method)	3.285
HB Acceptor	7
HB Donor	0
TPSA	48.39

| Results

Physicochemical Property

Property	Predicted values	Suggestions	Meaning & Preference	Reference
LogS (Solubility)	-4.301 log mol/L (20.031 µg/mL)	> 10 µg/ml	• Optimal: higher than -4 log mol/L<br>• <10 µg/mL: Low solubility; 10–60 µg/mL: Moderate solubility; >60 µg/mL: High solubility	• Book: ISBN: 9787562832287. pp. 14<br>• J PHARMACOL TOX MET. 2000, 44 (1), 235–249;
$LogD_{7.4}$ (Distribution Coefficient D)	2.872	1~5	• < 1: Solubility high; Permeability low by passive transcellular diffusion; Permeability possible via paracellular if MW < 200; Metabolism low.<br>• 1 to 3: Solubility moderate; Permeability moderate; Metabolism low.<br>• 3 to 5: Solubility low; Permeability high; Metabolism moderate to high.<br>• > 5: Solubility low; Permeability high; Metabolism high.	• Methods and principles in medicinal chemistry 18 (pp. 21–45). Weinheim: Wiley-VCH.

Property	Predicted values	Suggestions	Meaning & Preference	Reference
LogP (Distribution Coefficient P)	3.285	0~3	• Optimal: 0< LogP <3<br>• LogP <0: poor lipid bilayer permeability.<br>• LogP >3: poor aqueous solubility.	• Book: ISBN: 3-906390-22-5. pp. 127–182.

Absorption

Property	Predicted values	Probability	Suggestions	Meaning & Preference	Reference
Papp (Caco-2 Permeability)	-4.692 cm/s		> -5.15 cm/s	Optimal: higher than -5.15 Log unit or -4.70 or -4.80	• J CHEM INF MODEL. 2016, 56 (4), pp 763–773.
Pgp-inhibitor	+	0.5		• The Pgp-inhibitor & non-inhibitor classification criteria refers the reference.	• J CHEM INF MODEL. 2010. 50(6): p. 1034-1041.<br>• J MED CHEM. 2011. 54(6): p. 1740-1751.
Pgp-substrate	+	0.651		• More likely to be a Pgp substrate: N+O ≥ 8; MW > 400; Acid with pKa > 4<br>• More likely to be a Pgp non-substrate: N+O ≤ 4; MW < 400; Acid with pKa < 8	• J DRUG TARGET. 11, 391–406.
HIA (Human Intestinal Absorption)	+	0.699		• ≥30%: HIA+; <30%: HIA-	• RSC ADV. 2017, 7, 19007-19018
F (20% Bioavailability)	+	0.674		• ≥20%: F20+; <20%: F20-	• MOL PHARMACEUT, 2011. 8(3): p. 841-851<br>• J PHARMACEUT BIOMED, 2008. 47(4): p. 677-682.
F (30% Bioavailability)	+	0.629		• ≥30%: F30+; <30%: F30-	• MOL PHARMACEUT, 2011. 8(3): p. 841-851<br>• J PHARMACEUT BIOMED, 2008. 47(4): p. 677-682.

Distribution

Property	Predicted values	Probability	Suggestions	Meaning & Preference	Reference
PPB (Plasma Protein Binding)	67.433 %		90%	• Significant with drugs that are highly protein-bound and have a low therapeutic index.	• ISBN: 978-0-1236-9520-8. pp. 194

Property	Predicted values	Probability	Suggestions	Meaning & Preference	Reference
VD (Volume Distribution)	1.239 L/kg		0.04~20 L/kg	• Optimal: 0.04-20L/kg;<br>• Range: <0.07L/kg: Confined to blood, Bound to plasma protein or highly hydrophilic; 0.07-0.7L/kg: Evenly distributed; >0.7L/kg: Bound to tissue components (e.g., protein, lipid),highly lipophilic.	• Book: ISBN: 9787562832287. pp. 174<br>• Book: ISBN: 978-0-1236-9520-8. pp. 229
BBB (Blood–Brain Barrier)	++	0.817		• BB ratio >=0.1: BBB+; BB ratio <0.1: BBB-<br>• These features tend to improve BBB permeation: H-bonds (total) < 8–10; MW < 400–500; No acids.	• J NEUROCHEM. 70, 1781–1792

Metabolism

Property	Predicted values	Probability	Meaning & Preference	Reference
P450 CYP1A2 inhibitor	+	0.546	• Molecules that labeled inhibitor in PubChem BioAssay were regarded as inhibitor.	• NAT BIOTECHNOL. 2009, 27(11): 1050-1055.<br>• BIOINFORMATICS. 2013, 29(16): 2051-2052.
P450 CYP1A2 Substrate	+	0.61	• Molecules that labeled substrate in PubChem BioAssay were regarded as substrate.<br>• Characteristics of CYP1A2 substrate: 0.08< LogP <3.61; Planar amines and amides	• NAT BIOTECHNOL. 2009, 27(11): 1050-1055.<br>• BIOINFORMATICS. 2013, 29(16): 2051-2052.
P450 CYP3A4 inhibitor	---	0.177	• Molecules that labeled inhibitor in PubChem BioAssay were regarded as inhibitor.<br>• Strategies to Reduce CYP3A4 Inhibition: Decrease the lipophilicity (LogD $_{7.4}$); Add steric hindrance to the heterocycle para to the nitrogen; Add an electronic substitution (e.g., halogen) that reduces the pKa of the nitrogen.	• NAT BIOTECHNOL. 2009, 27(11): 1050-1055.<br>• BIOINFORMATICS. 2013, 29(16): 2051-2052.
P450 CYP3A4 substrate	+	0.67	• Molecules that labeled substrate in PubChem BioAssay were regarded as substrate.<br>• Characteristics of CYP3A4 substrate: 0.97< LogP <7.54; Large molecules	• NAT BIOTECHNOL. 2009, 27(11): 1050-1055.<br>• BIOINFORMATICS. 2013, 29(16): 2051-2052.<br>• ISBN: 978-0-1236-9520-8. pp. 162

Property	Predicted values	Probability	Meaning & Preference	Reference
P450 CYP2C9 inhibitor	---	0.136	• Molecules that labeled inhibitor in PubChem BioAssay were regarded as inhibitor.	• NAT BIOTECHNOL. 2009, 27(11): 1050-1055. • BIOINFORMATICS. 2013, 29(16): 2051-2052.
P450 CYP2C9 substrate	-	0.37	• Molecules that labeled substrate in PubChem BioAssay were regarded as substrate. • Characteristics of CYP2C9 substrate: 0.89< LogP <5.18; Acidic (Nonionized)	• MOL INFORM. 2011. 30(10): p. 885-895. • J CHEM INF MODEL. 2013. 53(12): p. 3373-3383. • ISBN: 978-0-1236-9520-8. pp. 162
P450 CYP2C19 inhibitor	---	0.214	• Molecules that labeled inhibitor in PubChem BioAssay were regarded as inhibitor.	• NAT BIOTECHNOL. 2009, 27(11): 1050-1055. • BIOINFORMATICS. 2013, 29(16): 2051-2052.
P450 CYP2C19 substrate	+	0.639	• Molecules that labeled substrate in PubChem BioAssay were regarded as substrate.	• NAT BIOTECHNOL. 2009, 27(11): 1050-1055. • BIOINFORMATICS. 2013, 29(16): 2051-2052.
P450 CYP2D6 inhibitor	-	0.447	• Molecules that labeled inhibitor in PubChem BioAssay were regarded as inhibitor.	• MOL INFORM. 2011. 30(10): p. 885-895. • J CHEM INF MODEL. 2013. 53(12): p. 3373-3383.
P450 CYP2D6 substrate	-	0.412	• Molecules that labeled substrate in PubChem BioAssay were regarded as substrate. • Characteristics of CYP2D6 substrate: 0.75< LogP <5.04; Basic (Ionized)	• MOL INFORM. 2011. 30(10): p. 885-895. • J CHEM INF MODEL. 2013. 53(12): p. 3373-3383. • ISBN: 978-0-1236-9520-8. pp. 162

Elimination

Property	Predicted values	Suggestions	Meaning & Preference	Reference
$T_{1/2}$ (Half Life Time)	2.008 h	> 0.5 h	• Range: >8h: high; 3h< Cl < 8h: moderate; <3h: low	• ISBN: 978-0-1236-9520-8. pp. 236
CL (Clearance Rate)	1.867 mL/min/kg		• Range: >15 mL/min/kg: high; 5mL/min/kg< Cl < 15mL/min/kg: moderate; <5 mL/min/kg: low	• ISBN: 978-0-1236-9520-8. pp. 236

Toxicity

Property	Predicted values	Probability	Suggestions	Meaning & Preference	Reference
hERG (hERG Blockers)	++	0.775		• Where molecules with IC50 < 40 µM were regarded as blockers.<br>• Features may lead to hERG blocker:<br>◦ A basic amine (positively ionizable, pKa >7.3).<br>◦ Hydrophobic/lipophilic substructure(s) (ClogP >3.7).<br>◦ Absence of negatively ionizable groups or oxygen H-bond acceptors.	• TRENDS PHARMACOL SCI. 2005, 26(3): 119-124<br>• ISBN: 978-0-1236-9520-8. pp. 213<br>• MOL PHARM. 2016, 13(8):2855–2866
H-HT (Human Hepatotoxicity)	+	0.594		• The H-HT positive(+) & negative(-) classification criteria refers the reference.	• CHEM RES TOXICOL, 2016, 29(5): 757-767.
AMES (Ames Mutagenicity)	---	0.228		• Ames positive(+) & negative(-): significantly induces revertant colony growth at least in one out of usually five strains, otherwise, negative.	• J CHEM INF MODEL. 2012, 52(11): 2840-2847.
SkinSen (Skin sensitization)	-	0.4		• Sensitizer & Non-sensitizer: The (r)LLNA experimental value. (r)LLNA: (Reduced) local lymph node assay.	• TOXICOL APPL PHARM, 2015 , 284 (2) :262-272
LD50 (LD50 of acute toxicity)	2.678 -log mol/kg (840.827 mg/kg)		> 500 mg/kg	• Median lethal dose (LD50) usually represents the acute toxicity of chemicals.It is the dose amount of a tested molecule to kill 50 % of the treated animals within a given period.<br>• High-toxicity: 1~50 mg/kg; Toxicity: 51~500 mg/kg; low-toxicity: 501~5000 mg/kg.	• CHEM RES TOXICOL, 2009, 22 (12), pp 1913–1921<br>• J CHEMINFORMATICS, 2016 , 8 (1) :6
DILI (Drug Induced Liver Injury)	+	0.522		• The DILI positive(+) & negative(-) classification criteria refers the reference.	• J CHEM INF MODEL, 2015, 55(10) :2085-2093
FDAMDD (Maximum Recommended Daily Dose)	-	0.384		• The FDAMDD positive(+) & negative(-) classification criteria refers the reference.	• CHEMOMETR INTELL LAB, 2015, 146:494-502
